# Nationwide study on development and validation of a risk prediction model for CIN3+ and cervical cancer in Estonia

**DOI:** 10.1038/s41598-024-75697-3

**Published:** 2024-10-19

**Authors:** Anna Tisler, Andres Võrk, Martin Tammemägi, Sven Erik Ojavee, Mait Raag, Aleksandra Šavrova, Mari Nygård, Jan F. Nygård, Mindaugas Stankunas, Anda Kivite-Urtane, Anneli Uusküla

**Affiliations:** 1 Institute of Family Medicine and Public Health, University of Tartu, Ravila 19, 50411 Tartu, Estonia; 2 Johan Skytte Institute of Political Studies, University of Tartu, Tartu, Estonia; 3 Department of Health Sciences, Brock University, St Catharines, ON Canada; 4 Department of Computational Biology, University of Lausanne, Lausanne, Switzerland; 5 Estonian Health Insurance Fund, Tartu, Estonia; 6 North Estonia Medical Centre, Women’s Clinic, Tallinn, Estonia; 7 Cancer Registry of Norway, Norwegian Institute of Public Health, Oslo, Norway; 8 Department of Health Management, Lithuanian University of Health Sciences, Kaunas, Lithuania; 9 Institute of Public Health, Riga Stradins University, Riga, Latvia

**Keywords:** Cervical cancer, Prediction, High-grade lesions, Screening, Model, Health data, Cancer models, Cancer prevention, Cervical cancer

## Abstract

Transitioning to an individualized risk-based approach can significantly enhance cervical cancer screening programs. We aimed to derive and internally validate a prediction model for assessing the risk of cervical intraepithelial neoplasia grade 3 or higher (CIN3+) and cancer in women eligible for screening. This retrospective study utilized data from the Estonian electronic health records, including 517,884 women from the health insurance database and linked health registries. We employed Cox proportional hazard regression, incorporating reproductive and medical history variables (14 covariates), and utilized the least absolute shrinkage and selection operator (LASSO) for variable selection. A 10-fold cross-validation for internal validation of the model was used. The main outcomes were the performance of discrimination and calibration. Over the 8-year follow-up, we identified 1326 women with cervical cancer and 5929 with CIN3+, with absolute risks of 0.3% and 1.1%, respectively. The prediction model for CIN3 + and cervical cancer had good discriminative power and was well calibrated Harrell’s C of 0.74 (0.73–0.74) (calibration slope 1.00 (0.97–1.02) and 0.67 (0.66–0.69) (calibration slope 0.92 (0.84–1.00) respectively. A developed model based on nationwide electronic health data showed potential utility for risk stratification to supplement screening efforts. This work was supported through grants number PRG2218 from the Estonian Research Council, and EMP416 from the EEA (European Economic Area) and Norway Grants.

**Supplementary Information:**

The online version contains supplementary material available at 10.1038/s41598-024-75697-3.

## Introduction

Public health initiatives aim to eradicate cervical cancer by the year 2030. As of 2020, 22 (82%) European Union (EU) Member States had integrated population-based screening programs for cervical cancer into their National Cancer Control Plans^1^. While organized screening programs have been instrumental in reducing cervical cancer incidence and mortality, they often rely on a standardized, age-based strategy applied uniformly across the population^2^. The issues associated with a “one-size-fits-all” screening approach include suboptimal attendance, over-screening, and significant disparities in the utilization of cancer screening services^3^. These limitations underscore the urgent need for more effective, tailored screening programs that account for individual risk factors. Cervical cancer screening offers an excellent framework for personalized risk assessment due to well-established disease patterns and key risk factors: persistent high-risk HPV infection, age, sexual history, oral contraceptive use, smoking, and screening non-attendance^4^. Risk-stratified screening has emerged as a concept in which decisions to offer screening or the determination of screening frequency and modality (screening test(s)) are guided by accurate estimation of an individual’s risk of cancer^5^. Risk-based screening aims to optimize benefits (reducing cancer-related deaths) while minimizing potential harms (excess screenings, false positives, and overdiagnosis).

Over the past decade, numerous cervical cancer predictive models have been developed^6^, but a systematic review reveals persistent challenges, including methodological inconsistencies, limited population representativeness, and small sample sizes that hinder generalizability. Electronic health data-based models have the potential to address these issues. By offering a comprehensive and detailed view of individual health histories and current statuses, electronic health data could significantly enhance the accuracy of risk assessments. Additionally, the real-time availability of this data allows for frequent updates to models, ensuring they reflect the most current information and emerging health trends. The extensive scale of electronic health data also facilitates the inclusion of diverse patient populations in model training, improving generalizability and overcoming limitations related to sample size and population diversity. Thus, leveraging electronic health data could provide a valuable approach to overcoming the challenges faced by existing predictive models.

This study aimed to develop and validate a model that predicts and evaluates risk over an 8- and 5-year horizon of cervical intraepithelial neoplasia grade 3 or higher (CIN3+) and cancer in the adult female population using nationwide, linked electronic health data.

### Setting

In Estonia, an organized cervical cancer screening program utilizing Pap tests (cytology) every five years was initiated in 2006, targeting women aged 30–55 years. The participation rate in this screening program has been suboptimal, with attendance consistently falling below 50%^7^. Over recent decades, there has been a shift towards diagnosing cervical cancer at more advanced stages^8^. Approximately 90% of cervical cancer cases in Estonia have been detected outside of routine screening and through testing symptomatic women. This has had a minimal effect on the estimated age-standardized incidence rate of cervical cancer with 14.4 cases per 100,000 women during the period 2014–2018^9^ which is roughly twice as high as those estimated for Western Europe (6.8 per 100,000), Northern America (6.4 per 100,000), and Australia (6.0 per 100,000)^10^.

Despite nearly two decades of the national cervical cancer (CC) screening program, the CC incidence in Estonia remains one of the highest in Europe. Given the availability of comprehensive nationwide electronic health data, developing a risk prediction model for CC in Estonia is essential. Such a model could enhance the effectiveness of the screening program by identifying high-risk individuals who would benefit from more frequent and targeted screening, potentially improving early detection rates and reducing the overall burden of the disease. The HPV vaccination program commenced in 2009 for girls aged 12 to 14, and since 2024 the also includes boys.

## Methods

### Study design

In this retrospective modelling and internal validation study, data for model development and internal validation were derived from the following Estonian health registries: data from the Estonian Health Insurance Fund (EHIF)^11^, Estonian Cancer Registry (ECR), and Estonian Medical Birth Registry (EMBR) were employed (Supplementary Tables 1 and data source description). These are national health data sources that can be linked using unique personal identification codes. Data spanning from 2005 to 2012 were utilized to develop a risk-based model using routinely collected electronic health data. The eight-year period was chosen to ensure sufficient time for collecting and evaluating relevant predictors. Data on all women born in 1988 or earlier (aged ≥ 16 years on the 1st of January 2005) in the Estonian Health Insurance Fund (EHIF) were followed from the 1st of January 2005 until the 31st of December 2012.

The model was then validated over eight-year period (2013–2020), chosen to extend the national screening recommendation interval of five years. The model validation was made from Jan 1, 2013, to December 31, 2020, and the data excluded all women with a previous indication of cervical and uterine cancer.

**Fig. 1:**
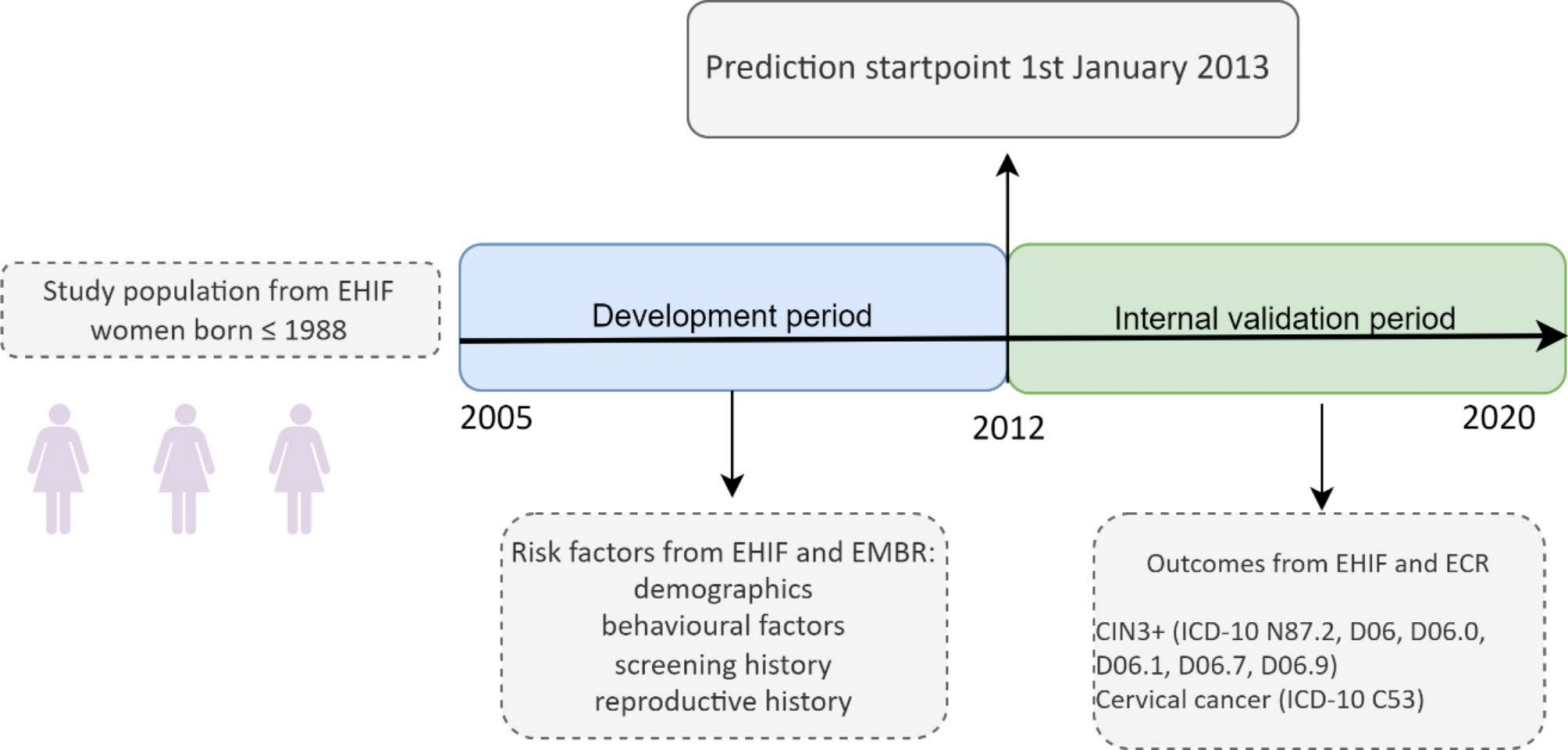
Risk prediction model workflow. *EHIF* Estonian Health Insurance Fund, *ECR* Estonian Cancer Registry, *EMBR* Estonian Medical Birth Registry, *CIN3* cervical intraepithelial neoplasia grade 3.

Data on medical/health history were supplemented with sociodemographic and reproductive history (Supplementary Table 1) and were employed to create a prediction model for two outcomes: CIN3 + and cervical cancer (Fig. 1). Model development and validation were performed following the clinical prediction rules and guidelines^12^.

### Data sources

Estonian Health Insurance Fund (EHIF)^11^ provides universal public health insurance since 2001 and covers > 95% of the Estonian population (95.8% of the female population aged 16 or older in 2021)^13^. EHIF maintains a comprehensive healthcare and prescription pharmaceutical database, including personal data (sex, year of birth) and healthcare utilization (services provided, date of service, primary and secondary diagnoses, inpatient and outpatient treatments) The diagnoses are presented using the International Classification of Diseases, Tenth Revision (ICD-10), while medical services are coded using the Nordic Medico-Statistical Committee (NOMESCO) codes. The prescriptions database contains detailed information about all prescribed and purchased medications and vaccines. Full electronic data from the EHIF have been available since 2005.

The Estonian health insurance system adheres to universal coverage principles, and individuals’ insurance status may change based on various factors, such as employment and residency status.

Estonian Cancer Registry (ECR) is a population-based registry in operation since 1978 containing complete and reliable registration of incident cancer cases. In Estonia, reporting cancer cases is compulsory for all physicians who treat and diagnose cancer. The validity of Estonian Cancer Registry data is at a favorable international level^14^.

Estonian Medical Birth Registry (EMBR^15^) was established in 1991 to collect data on all births in Estonia. All maternity units in Estonia are obliged to notify births to the EMBR. The notification form includes the personal identity number of the mother, and information about maternal socio-demographics, health behaviour and health before and during pregnancy. Data on births before 1991 are not available. The study omitted the years 1991–1994 due to incomplete records resulting from the gradual issuing of personal identification codes (PIC-s) to individuals in the early 1990s. Consequently, the analysis concentrated solely on complete records beginning from 1994 onward.

In Estonia, unique 11-digit PIC-s are assigned to all residents at birth or at the time of immigration. PIC as a single unique identifier is recorded accurately in all three data sources used in this study, enabling a straightforward and complete linkage of study population information between the registries.

### Study population

The study population consisted of all women born ≤ 1988 identified from the EHIF data. Women who died before 1 January 2013, or with no information regarding health insurance as a predictor during the development period (2005–2012) or validation period (2013–2020), or those considered not at risk of cervical cancer such as women with a history of cervical cancer or those who had undergone total hysterectomy, including uterine cancer were excluded from the risk model.

For the analysis, two cohorts were established: Cohort 1, which included all women born ≤ 1988, and Cohort 2, consisting of women born between 1977 and 1988. The rationale for developing a separate model for the younger cohort was based on the timing of the validation period, where these individuals would be entering the screening age. Furthermore, the birth registry data starting from 1994 for Cohort 2 is more comprehensive than that available for women in Cohort 1.

### Predictors (medical/health and reproductive history, socio-demographic characteristics)

The predictors incorporated were based on previous research on risk factors for cervical cancer^16,17^. For both cohorts (Cohort 1: all women; Cohort 2: younger women) the data on socio-demographics (year of birth, health insurance status), cervical cancer screening participation (PAP tests), systemic hormonal contraception use, data on diagnosed sexually transmitted infections (STIs) were derived from EHIF. Additional data on the number of births, smoking history (ever during the development period), and education from the EMBR. Variables definitions and data sources are provided in Supplementary Table 1.

### Modelling outcomes

Cervical intraepithelial neoplasia 3 or more severe cases (CIN3+) were primary outcomes, considered the most reliable surrogate marker for cervical cancer risk. Cervical cancer was considered a secondary outcome (Supplementary Table 1). We did not perform a formal sample size estimation as we utilized nationwide data on all CIN3 + and cervical cancer cases.

Our sample size (outcome counts) aligns well with the recommendation to have at least 10 events per variable, which minimizes bias and ensures predictive accuracy in Cox proportional hazards models^18^.

### Model derivation

The Cox proportional hazards model was employed to predict study outcomes up to 8 years post-development, with the index date set as January 1, 2013. Additionally, we predicted 5-year outcome risks using the same model, evaluating its performance within this interval following the national cervical cancer screening guidelines. The rationale for these specific timeframes is twofold: the 5-year interval aligns with Estonia’s recommended cervical cancer screening interval, while the 8-year interval reflects the potential extension of screening intervals. To select the predictors for the Cox models, we employed the Least Absolute Shrinkage and Selection Operator (LASSO) method (see results in Figs. 3 and 4), while using a 10-fold cross-validation approach. For every cohort and outcome combination, we identified the variables from the penalized Cox models in which the estimated lambda yielded the highest out-of-sample Harrell’s C statistic. For both outcomes, we present separate final Cox models for Cohort 1 (all women born ≤ 1988) and Cohort 2 (younger women born 1977–1988) as their coefficients and likelihood ratio test statistics (Supplementary Tables 4, 5), a total of four models are reported. Additionally, separate results for models that were fitted using all predictors and only those generated from EHIF data are reported in Supplementary Table 4.

We did not impute missing data due to their non-random absence. The rationale behind this approach was to construct a model that mirrors the actual real-world scenario. Missing data are frequently encountered in the context of routinely collected health information^19^, and such missingness often carries informative implications. We addressed this issue by including specific predictor variables that incorporate a category for ’Not available’ as one of the values (Table 1).

### Model performance

The statistical performance of risk prediction models was assessed by discrimination, calibration and clinical utility^20^. Our study employed 10-fold cross-validation for internal validation. This procedure allowed us to generate out-of-sample predictions for the linear index of a Cox model and predicted risks, facilitating the assessment of discrimination and calibration in our analysis. Discrimination (classification accuracy) was assessed using a time-dependent area under the receiver operating characteristic curves (AUROC), employing inverse censoring probability weighting over a 5 and 8-year timeframe. The developed model’s discriminatory performance was measured by Harrell’s C -statistic (ranging from 0 to 1, with value higher than 0.75 demonstrating useful discrimination^21^. 95% confidence intervals (CI) are provided. Calibration plots (Supplementary Figs. 3–14) in deciles were used to examine the agreement between model-predicted and observed probabilities and report calibration slopes. Finally, we evaluated the clinical utility of the prediction models using a decision curve analysis^22^. Net benefit serves as a metric to evaluate the pros and cons of using a model for clinical decision support and for conducting impact studies. We report a range of thresholds at which the model demonstrated a net benefit.

All analyses were conducted using SAS 9.3 (Cary, NC) and R 4.2.3 (https://cran.r-project.org/).

### Ethics

This study was approved by the ethical review board at the University of Tartu (protocol number: 3320/M-7, 21.12.2020) which waived the requirement to obtain informed consent. We followed the Transparent Reporting of a Multivariable Prediction Model for Individual Prognosis or Diagnosis (TRIPOD) checklist to ensure transparent reporting^23^. The whole research was performed in accordance with the relevant guidelines and regulations.

## Results

### Study cohort and development period (2005–2012)

**Fig. 2:**
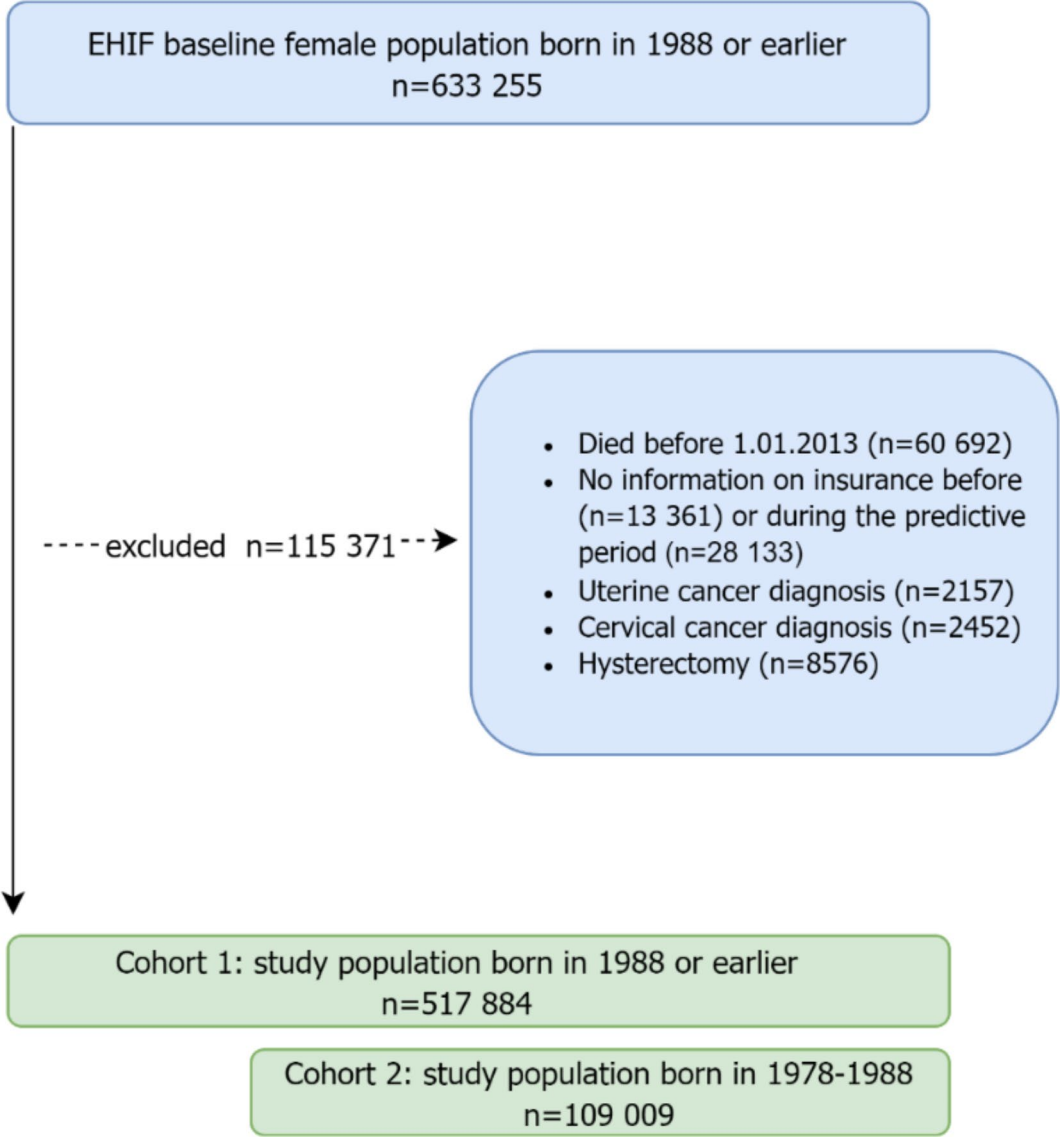
Flowchart for the study population during the 2005–2012 development period. Excluded individuals are counted only once.

Using the EHIF database we identified 633 255 women born ≤ 1988. Of those, 18.2% (*n* = 115 371) were excluded from the analysis (Fig. 4). Our study sample contained data on 517 884 women born in 1988 or earlier (Cohort 1) and those born between 1977 and 1988 (21.0%, *n* = 109 009) were identified as Cohort 2 (Fig. 2).

The participants’ characteristics during the development period 2005–2012 are presented in Table 1. The mean age of the study population on December 31, 2012, was 54.2 (range 25–109 years) and 30.03 (range 25–36 years) years in Cohort 1 and Cohort 2 respectively. In Cohort 1, 41.8% had no PAP test for 8 years (PAP test coverage = 0), HIV, HPV (genital warts), and genital chlamydia infections were diagnosed in 0.2%, 0.9%, and 2.2% of women respectively.

**Table 1:** Study population characteristics up to the validation period.

	Cohort 1 (women born ≤ 1988)	Cohort 2 (women born 1977–1988)
Total	517 884 (100%)	109 009 (100%)
Age, mean (SD), 31.12.2012	54.2 (17.9)	30.3 (3.5)
Year of birth		
… 1932	43,980 (8.5%)	
1933–1946	98,551 (19%)	
1947–1956	90,975 (17.6%)	
1957–1966	88,290 (17.0%)	
1967–1976	87,079 (16.8%)	
1977–1982	52,300 (10.1%)	52,300 (48.0%)
1983–1988	56,709 (11%)	56,709 (52.0%)
Neoplasia (severest diagnosis)		
CIN 1 (N87.0)	9332 (1.8%)	4449 (4.1%)
CIN2 (N87.1) or CIN unclear (N87, N87.9)	11,188 (2.2%)	4907 (4.5%)
CIN3 (N87.2) or carcinoma in situ, CIS(D06)	3622 (0.7%)	1483 (1.4%)
None	493,742 (95.3%)	98,170 (90.1%)
Contraceptive coverage (systemic hormonal)^a^	0.07 (SD = 0.17)	0.19 (SD = 0.25)
0	393,162 (75.9%)	37,069 (34%)
< 0.10	48,307 (9.3%)	22,645 (20.8%)
0.10–0.40	41,788 (8.1%)	27,024 (24.8%)
0.40–0.60	16,330 (3.2%)	11,366 (10.4%)
0.60-1	18,297 (3.5%)	10,905 (10%)
PAP test coverage^a^	0.29 (SD = 0.31)	0.43 (SD = 0.31)
0	216,363 (41.8%)	19,092 (17.5%)
< 0.20	41,820 (8.1%)	10,234 (9.4%)
0.20–0.40	92,977 (18%)	23,890 (21.9%)
0.40–0.80	117,520 (22.7%)	39,307 (36.1%)
0.80-1.00	49,204 (9.5%)	16,138 (14.8%)
Proportion of period covered by health Insurance^a^	0.96 (SD = 0.13)	0.93 (SD = 0.16)
0.01–0.50	14,009 (2.7%)	4495 (4.1%)
0.50–0.80	19,632 (3.8%)	7229 (6.6%)
0.80–0.99	53,006 (10.2%)	23,468 (21.5%)
1	431,237 (83.3%)	73,817 (67.7%)
Number of births, mean (SD)	0.557 (SD = 1.061)	1.001 (SD = 0.993)
0	41,565 (8.0%)	41,565 (38.1%)
1	51,171 (9.9%)	34,757 (31.9%)
2	59,851 (11.6%)	25,748 (23.6%)
3+	33,420 (6.5%)	6939 (6.4%)
NA	331,877 (64.1%)	0
Number of abortions, mean (SD)^a^		0.390 (SD = 0.803)
0	467,386 (90.2%)	80,485 (73.8%)
1	35,858 (6.9%)	19,557 (17.9%)
2	9899 (1.9%)	5846 (5.4%)
3+	4741 (0.9%)	3121 (2.9%)
Smoking (ever)		
Yes	13,130 (2.5%)	7528 (6.9%)
No	145,525 (28.1%)	76,736 (70.4%)
No data	359,229 (69.4%)	24,745 (22.7%)
Education		
Primary	16,427 (3.2%)	11,290 (10.4%)
Secondary	92,245 (17.8%)	41,841 (38.4%)
Tertiary	57,488 (11.1%)	35,152 (32.2%)
No data	351,724 (67.9%)	20,726 (19%)
HPV^b^	4638 (0.9%)	2991 (2.74%)
HIV	1096 (0.21%)	878 (0.81%)
Genital chlamydia	11,280 (2.18%)	7840 (19%)
Other STD	56,855 (10.98%)	24,893 (22.84%)

^a^Continuous variables were discretized into categorical forms exclusively for the purpose of data visualization and presentation.

^b^Based on genital warts diagnosis (A63.0) in the billing data.

### Study outcomes during the validation period (2013–2020)

In Cohort 1 over the validation period of 8 years, a total of 1326 cervical cancer cases were diagnosed among 517,884 women (cumulative incidence of 0.26%). With 3,897,120 person-years of follow-up, the incidence rate was 34 per 100,000 person-years. The total number of CIN3 + cases identified was 5929 yielding a cumulative incidence of 1.14% and an incidence rate of 152 per 100,000 person-years (Supplementary Table 2). In the validation period, the mean age at diagnosis was 59 years for women with invasive cervical cancer and 46 years for women with CIN3+. In Cohort 2 during follow-up of 857 439 person-years CIN3 + and cervical cancer were diagnosed in 2697 (2.5%) and 172 (0.16%) women (incidence rates being 314 and 21 per 100,000 person-years respectively) (Supplementary Table 2). Similar data for the 5-year horizon is reported in Supplementary Table 3.

### Cohort 1

**Fig. 3:**
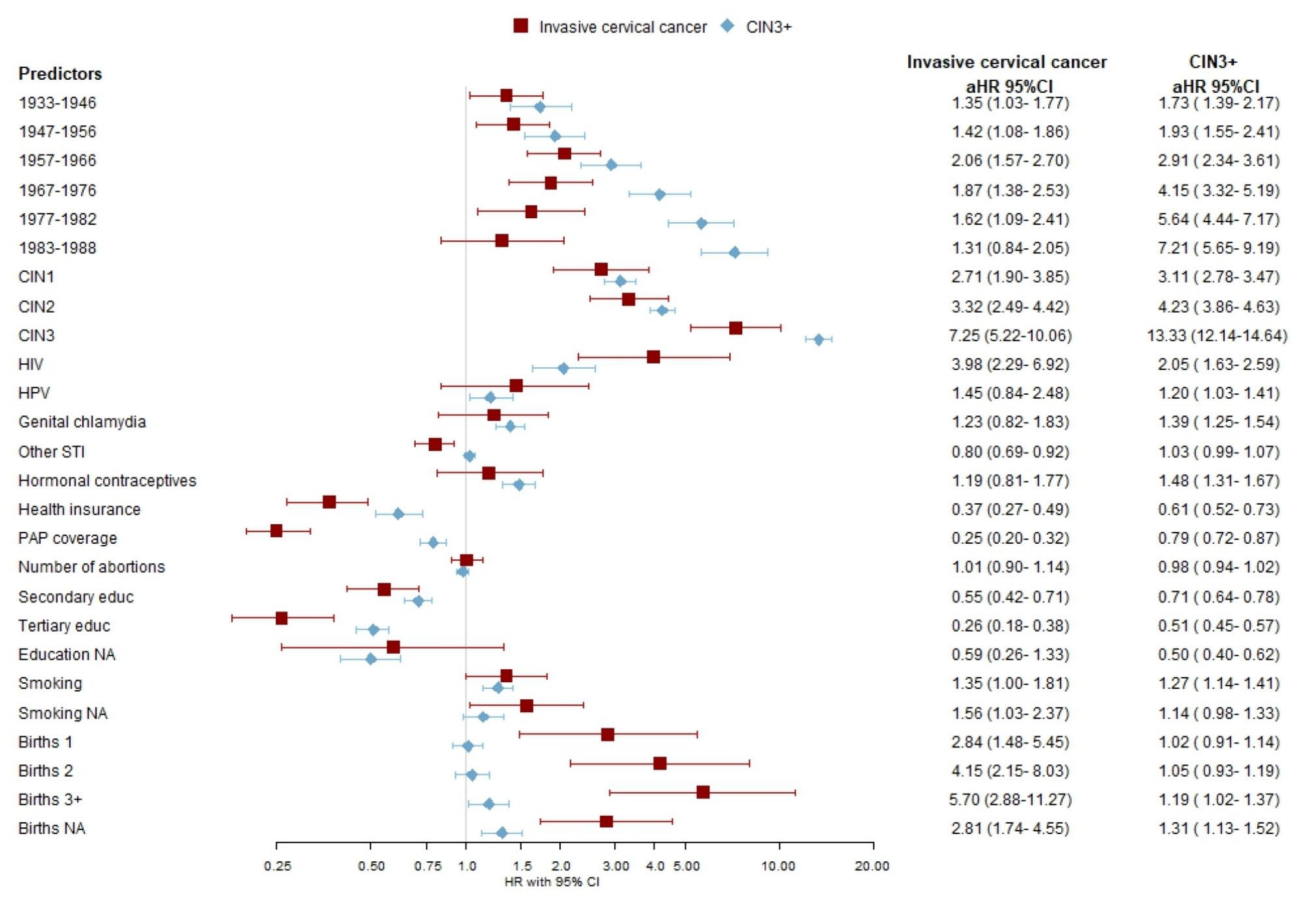
Adjusted hazard ratio (aHR) and 95% CI of invasive cervical cancer and high-grade precancerous cervical lesions (CIN3+) in relationship to risk factors using electronic health data among those born ≤ 1988 (Cohort 1) in Estonia, 2013–2020.

**Fig. 4:**
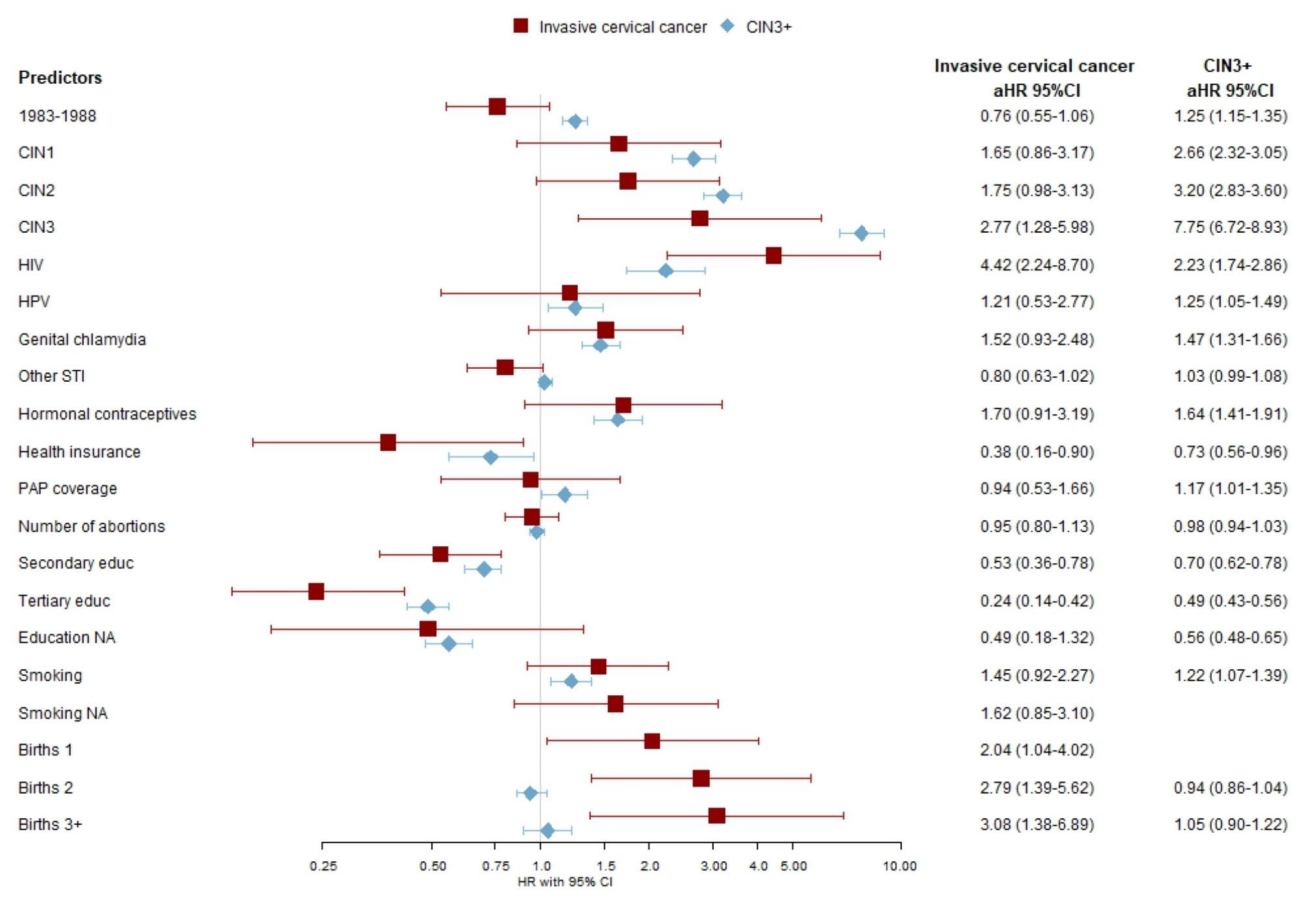
>Adjusted hazard ratios (aHR) and 95% CI of invasive cervical cancer and high-grade precancerous cervical lesions (CIN3+) in relationship to risk factors from electronic health records among those born in 1977–1988 (Cohort 2) in Estonia, 2013–2021.

Adjusted HR from multivariable models fitted separately for cervical cancer and CIN3 + are illustrated in Figs. 3 and 4. In the final model for Cohort 1 a higher risk of CIN3 + was observed for those diagnosed previously with HIV, HPV and genital chlamydia. In addition, long-term hormonal contraceptive use, younger age, smoking and previously diagnosed cervical neoplasias were significant predictors with the strongest associations noted for those with previous CIN3 diagnoses (HR 13.33; 95% CI 12.14–14.64) and those born in 1983–1988 (HR 7.21; 95% CI 5.65–9.19). Having health insurance and a history of PAP testing were protective factors. The risk for invasive cervical cancer was significantly increased among those with previous cervical neoplasias, especially CIN3, those living with HIV and the increasing number of births. PAP test coverage, being insured and higher education were inversely associated with cancer risk.

### Cohort 2

For the younger cohort previous HIV, HPV, long-term contraceptive use, genital chlamydia infections and smoking had significantly increased risk for CIN3+. Tertiary education and health insurance coverage were inversely associated with CIN3+. In the case of cervical cancer as an outcome following risk predictors were identified: history of CIN3, number of births, and HIV. Similar to the CIN3+, having health insurance and tertiary education were protective factors.

### Model performance

All model performance measures were evaluated using out-of-sample predictions from the cross-validation procedure.

#### Cohort 1

In the final model with CIN3 + as an outcome, all available predictors were included for Cohort 1. The discriminative capability of the CIN3 + model using Cox regression was good, with a cross-validated Harrell’s C index of 0.74 (95%CI: 0.73–0.74) (Table 2). The Cox model for CIN3 + showed excellent agreement between predicted and observed risks overall, with a calibration slope of 1.0 (95%CI 0.97–1.02). It’s worth noting that the model’s performance diminishes over time, with an AUROC of 0.74 for the 5-year risk, compared to an AUROC of 0.72 for the 8-year risk. The cervical cancer model showed good discrimination with a cross-validated Harrell’s C index of 0.67 (95% CI 0.66–0.69) and excellent calibration (Table 2).

#### Cohort 2

The Cox model for CIN3 + exhibited acceptable discriminative performance for Cohort 2, with a cross-validated Harrell’s C index of 0.68 (95% CI 0.67–0.69). The prediction accuracy decreased with a longer period 5-year AUROC 0.71 vs. 8 years AUROC 0.68 of prediction (Table 2). The cervical cancer model demonstrated satisfactory performance with a cross-validated Harrell’s C index of 0.67 (95% confidence interval: 0.63–0.71), exhibiting some degree of underestimation likely attributable to the limited frequency of events within the cohort studied (Table 2).

### Clinical utility

**Fig. 5:**
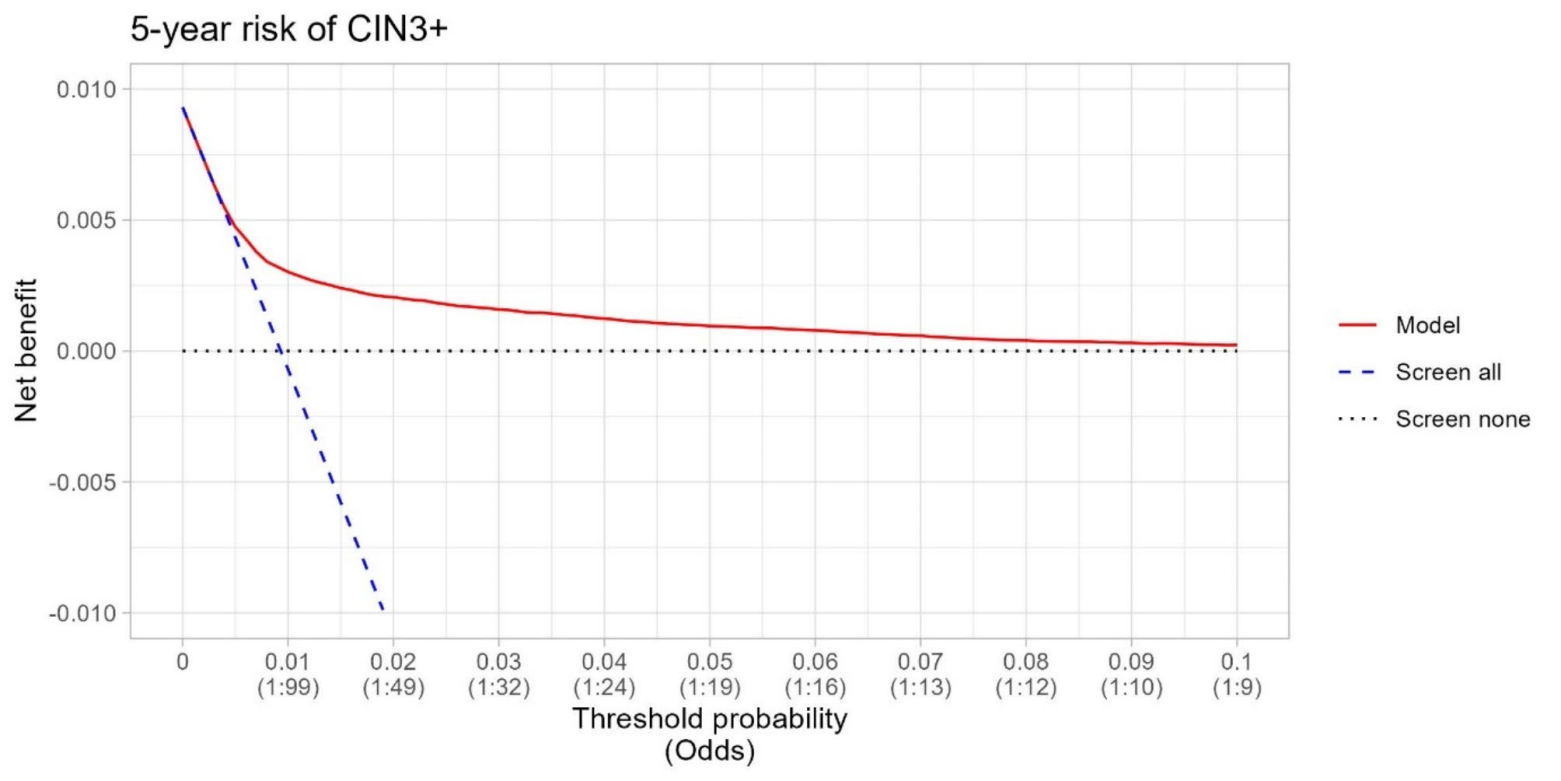
Decision curve analysis at 5 years for the CIN3 + risk prediction model among women aged 30–65 y using LASSO-based Cox.

**Fig. 6:**
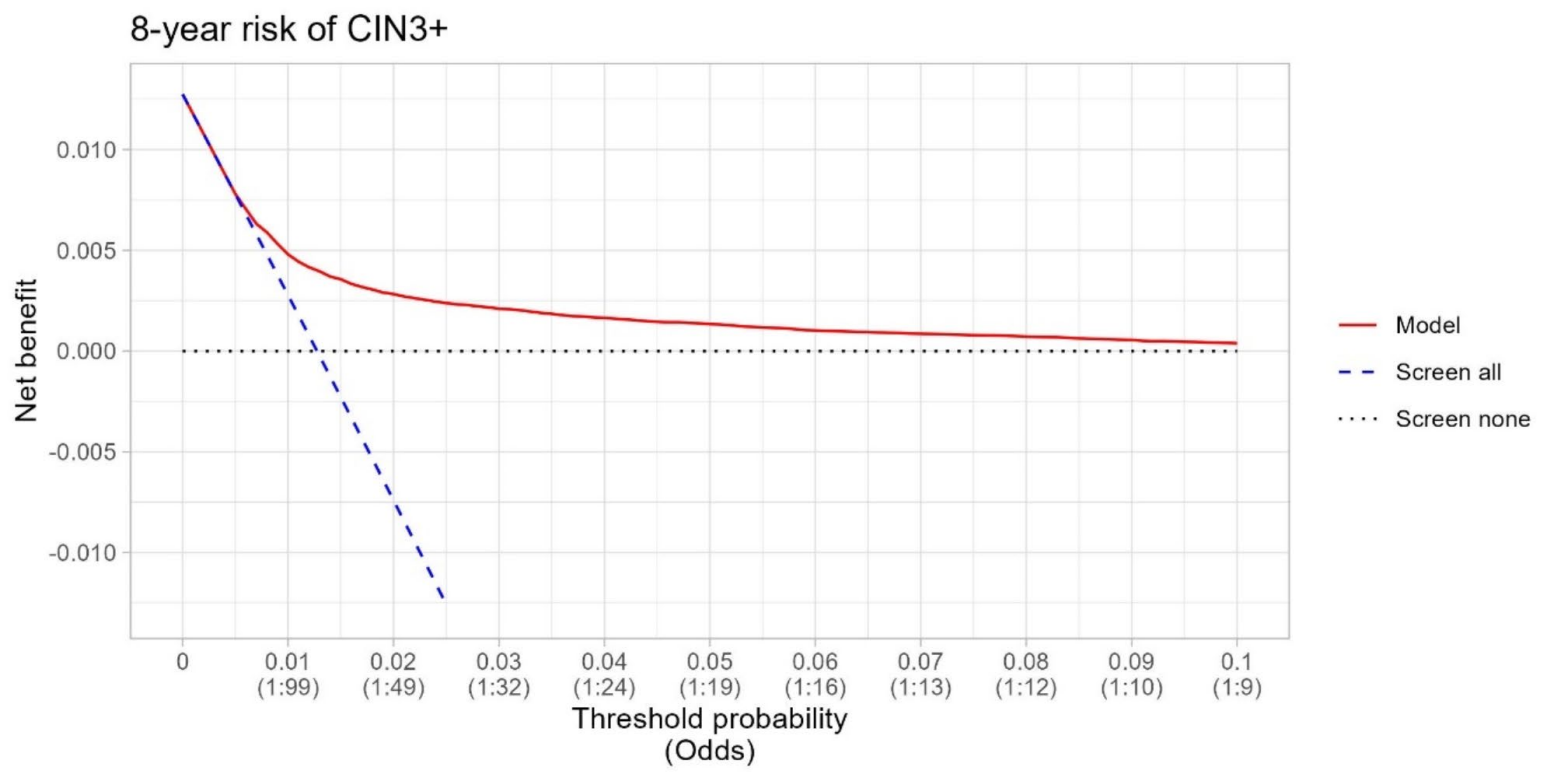
Decision curve analysis at 8 years for the CIN3 + risk prediction model among women aged 30–65 y using LASSO-based Cox model.

Decision curve analysis demonstrated the advantages of employing the Cox proportional hazards model for forecasting the 5 and 8-year risk of CIN3 + and cervical cancer for women aged 30–65 (Figs. 5 and 6 and Supplementary Figs. 1, 2). The decision curve of our CIN3 + model indicated that the use of the prediction model would be more beneficial than screening all and screening none at the threshold probabilities between 0.5% and 7% (Figs. 5 and 6). The most favorable net benefit was observed at thresholds of 0.009 (0.9%) for the 5-year risk and 0.013 (1.3%) for the 8-year risk compared to screening all.

**Table 2:** Performance of LASSO-based Cox regression models for CIN3 + and cervical cancer prediction.

Sample	Cohort 1		Cohort 2	
Outcome (95% CI)	Cancer	CIN3+	Cancer	CIN3+
Harrell’s C	0.68 (0.67–0.69)	0.74 (0.73–0.75)	0.72 (0.68–0.76)	0.69 (0.68–0.70)
Harrell’s C cross-validated	0.67 (0.66–0.69)	0.74 (0.73–0.74)	0.67 (0.63–0.71)	0.68 (0.67–0.69)
AUROC 5 years	0.66	0.74	0.67	0.71
AUROC 8 years	0.68	0.72	0.67	0.68
Observed/expected 5 years	1.00 (0.94–1.07)	1.01 (0.98–1.04)	1.00 (0.83–1.19)	1.00 (0.95–1.04)
Observed/expected 8 years	1.00 (0.95–1.06)	1.03 (1.00–1.01)	1.00 (0.86–1.16)	1.00 (0.96–1.04)
Calibration slope	0.92 (0.84–1.00)	1.00 (0.97–1.02)	0.76 (0.59–0.93)	0.99 (0.94–1.03)

## Discussion

In this study we have used nationwide health and registries data, to derive and internally validate a risk prediction model for estimating the risk of cervical precancerous lesions and cancer. Our study constitutes a noteworthy contribution to the knowledge base surrounding the utilization of national electronic health data for the establishment of risk-based screening^6^. Based on observed performance and comparing models to screen approach using decision curve analysis, this study has demonstrated the benefits of using a prediction modeling approach over decision rules based on a one-size-fits-all approach. This indicates their potential utility in informing screening programs for the target population. According to the prediction rules the decision curve analysis rationale of moving to external validation and no threshold are chosen at this point.

Our model is well-calibrated for this nationwide cohort of diverse women, as evidenced by the similarity between observed and predicted risks (Table 2). It demonstrates acceptable discrimination, accurately distinguishing between women with and without CIN3 + or cervical cancer (AUROC of 0.72 and 0.68 for an 8-year risk of CIN3 + and cancer respectively). In a Swedish study using machine learning to predict cervical cancer via electronic health records, the model’s performance worsened with longer prediction periods^24^. We observed a marginal decrease in discrimination from the 5- to 8-year prediction period. This highlights the model’s overall robustness and its feasibility for predicting CIN3 + risk over extended screening intervals, surpassing the current 5-year screening guidelines. There are no universally agreed-upon or recommended probability thresholds to guide (risk-based) cervical cancer screening. The recommendations in the latest cancer precursors management guidelines vary surveillance intervals based on the 5-year CIN3 + risk^25^ These intervals range from returning for testing in 1 year (for a risk of ≥ 0.55%) to returning in 5 years (for a risk of < 0.15%). While our model demonstrates observable net benefits across a range of threshold probabilities (spanning from 0.5 to 7.0%), the highest net benefit occurs at the 5-year risk of 0.9% and the 8-year risk of 1.3% for CIN3+. These results are informative and fall within the high-risk category for CIN3+. The determination of an appropriate probability threshold for risk stratification in cancer screening practice should consider local epidemiological risk factor profiles, the structure of the healthcare system, and available resources within the local context.

We opted to create new models instead of validating or updating existing ones due to variations in target populations, measurement procedures, changes over time and access to the nationwide health data. Although the models promise potentially efficient estimation of outcomes, cervical cancer prediction model development and validation remains in its infancy, grappling with the challenges of refining its algorithms and data sources^26^. Following internal validation, external validation, and randomized controlled trials are essential steps to further validate the model’s performance across diverse populations and settings, ensuring robustness and generalizability in clinical practice. Risk-based models must be regularly updated and refined using current and comprehensive datasets to remain useful and effectively address evolving clinical practices and risk factors (such as HPV genotypes and vaccination status)^27^.

By achieving performance comparable to individual cancer-specific risk prediction algorithms, the use of standardized and routinely collected electronic data offers a practical approach applicable to the entire population^28^. Importantly, this method allows for timely updates, accommodating changes in cervical cancer risk (as well as other cancers) over time.

Current prediction models for cervical cancer face several challenges, including methodological heterogeneity and limited representativeness of the general population. Furthermore, existing studies often suffer from small sample sizes. Although traditional regression models^29–31^ and machine learning approaches^32–34^ have been employed, there is considerable variability in the methodologies used. The minimally required AUC is still unknown, but studies on prediction models in related fields have suggested that an AUC of at least 0.8 is needed in more risk-based strategies^35^ To achieve this level of performance, larger study samples and the incorporation of additional predictors are essential. Expanding research efforts to address these needs will be crucial for developing robust and reliable cervical cancer screening models.

Our study had limitations. Health care and registry data often do not capture all pertinent predictors (i.e. data on a number of sexual partners, genetic risk markers or polygenic risk scores and type-specific HPV infection data were not available for us)^16^. The limited number of cancer cases in the dataset and the omission of temporal changes in predictor variables might have adversely impacted predictive performance. However, the former issue was mitigated by using the composite outcome of CIN3 + as the primary focus of the analysis. Also, the study population was predominantly of European descent, which might affect the generalizability of the model to other populations with different average risks. Internal validation yielded robust results, our model needs to be externally validated. Our model exhibited robust performance during internal validation, underscoring the need for external validation in new populations and potentially varied settings. This includes countries with similar healthcare systems and electronic health record infrastructures, such as those in the Baltic and Nordic regions.

Our study approach had several strengths. Our statistical analysis was informed by previously conducted risk-based models and was following clinical prediction rules. The LASSO method for selecting predictors penalizes model coefficients for over-optimism, creating a model that is more likely to exhibit consistent performance in new populations^36^. Using routinely collected health data offers multiple advantages. It leverages real healthcare experiences, is cost-effective, spans a wide timeframe for analyzing patient outcomes, and encompasses a diverse patient population. Last but not least, owing to the nature of administrative and registry data, such an approach could be applied to other cancers. The ability to update data promptly is crucial in the context of cervical cancer, given the changing risks throughout the life course^37^ By studying both cervical cancer and CIN3 predictions, we derived more comprehensive results, identifying individuals at various risk stages and enabling tailored intervention strategies.

## Conclusion

In this study, we derived and internally validated a model to predict the risk of cervical high-grade lesions and cancer 5- and 8 years ahead in a large, population-based nationwide cohort. Study results report good discrimination and calibration ability of the developed model. Findings suggest that electronic health data can be leveraged for risk assessment to inform screening efforts for cervical (pre)cancer at the population level.

## Electronic supplementary material

Below is the link to the electronic supplementary material.

Supplementary Material 1

## Data Availability

The datasets generated and/or analyzed during this study are not publicly available due to privacy and ethical restrictions to access the medical data, but they can be obtained from the corresponding author upon reasonable request.
