# Supplementary material for: Nationwide study on development and validation of a risk prediction model for CIN3+ and cervical cancer in Estonia
Source: Sci Rep. 2024 Oct 19;14:24589. doi: 10.1038/s41598-024-75697-3 (PMC11490536; doi:10.1038/s41598-024-75697-3)
Supplement: Supplementary file 1 — Supplementary Material 1 [file 41598_2024_75697_MOESM1_ESM.doc]

**Supplement Table 1. Definitions and encoding of predictors and outcomes**

**Predictors, data sources and definitions for the analysis of CIN3+ and cervical cancer (i.e. testing whether those variables predict diagnosis of CIN and CC) included established and suspected risk factors for CINs and CC. The following risk factors were therefore used (with the ICD-10 code in brackets when indicated):**

| **Predictors** | | | | |
| --- | --- | --- | --- | --- |
| Database | Variable | | Definition | |
| Estonian Health Insurance Fund (EHIF) | Year of birth | | As indicated in the health insurance data | |
|  | Presence of CIN1 (mild dysplasia) | | At least one medical bill with diagnosis code N87.0 in the observation period | |
|  | Presence of CIN2 (moderate dysplasia) | | At least one medical bill with diagnosis code N87.1 (or unclear diagnosis with N87, N87.9) in the observation period | |
|  | Presence of CIN3 (severe dysplasia or carcinoma in situ) | | At least one medical bill with diagnosis code N87.2 or D06, D06.0, D06.1, D06.7, D06.9 in the observation period | |
|  | Number of abortions | | Abortions are derived from ICD-10 diagnoses (O01, O02, O03, O04, O05, O06, O07, and O08) related to abortions on medical bills. Bills within 60 days are assigned to the same event.  . | |
|  | HIV | | At least one medical bill with diagnosis code B20, B21-24, Z21, R75, F02.4 | |
|  | Genital chlamydia | | At least one medical bill with diagnosis code A55-56 | |
|  | Other Sexually transmitted infections (STI) | | At least one bill with one of the following ICD-10 diagnosis (syphilis: A50, A51, A52, A53, gonorrhea: A54, trichomoniasis A59, anogenital herpes A60, other: A57, A58, A63, A64 | |
|  | HPV | | A63.0 | |
|  | PAP test coverage 2005-2012 | | Using specific service codes. Each test is assumed to cover 5 years. Overlapping periods are merged.  Coverage is defined as a fraction of days between 1.1.2005- 31.12.2012 covered by tests. (Details are available from authors) | |
|  | Contraceptive use | | Relative coverage with contraceptives is defined as a fraction of days between 1.1.2005-31.12.2012 covered by oral, vaginal, transdermal and implant hormonal contraceptives. Type of contraceptive and days covered are taken from the prescriptions.  Intrauterine devices are excluded.  (Details are available form authors) | |
|  | Presence of health insurance | | Fraction of biannual time points between 2005-2012 and 2013-2020 when person is covered by public health insurance. | |
| EHIF and Estonian Cancer Registry (ECR) | Presence of cervical cancer | | At least one medical bill with one of the diagnosis codes C53, C53.0, C53.1, C53.8, C53.9 in the EHIF registry or in the observation period. These women are all dropped from the analysis.  Presence in the cancer registry with the diagnosis C53 in the observation period. These women are all dropped from the analysis.  In the cancer outcome: presence in the cancer registry with the diagnosis C53 in the follow-up period.  In the CIN3+ outcome: presence either in the cancer registry with the diagnosis C53 or at least one bill with C53, C53.0, C53.1, C53.8, C53.9 in the EHIF registry in the follow-up period. (In addition to CIN3 codes N87.2 or D06, D06.0, D06.1, D06.7, D06.9 in the follow-up period.) | |
| Estonian Medical Birth Registry (EMBR) | smoking | | At least one record of smoking during pregnancy in the observation period | |
|  | education | | Most recent self-reported education categorised into three levels | |
|  | number of births | | Linked directly from the medical birth registry | |
| Outcomes | | | | |
| Cervical cancer | | EHIF and ECR | | Were identified by applying the specific ICD-10 codes (C53, C53.0, C53.1, C53.8, and C53.9). Cancer cases were flagged if at least one of the following conditions was satisfied: (1) the presence of an inpatient bill (2) a medical claim from an oncologist within the EHIF (Estonian Health Insurance Fund) records, (3) the presence of at least three medical claims from outpatient clinics or (4) an ECR (Estonian Cancer Registry) record indicating a diagnosis of cervical cancer. The earliest date of diagnosis was considered in the analysis for both outcomes. |
| CIN3+ | | EHIF | | In addition to cervical cancer definition provided, CIN3+ contained neoplasia diagnoses based on ICD-10 codes N87.2, D06, D06.0, D06.1, D06.7, D06.9 from at least one EHIF medical bill. |

**Supplement Table 2.** **Total and subgroup-specific number of women with CIN3+ and cervical cancer during the prediction period 2013-2020 (8-year timeframe)**

|  | **Cohort 1 (women born ≤1988)** | | | **Cohort 2 (women born 1977-1988)** | | |
| --- | --- | --- | --- | --- | --- | --- |
| Variable | N | CIN3+, rate per 100 000 person years | Cervical cancer, rate per 100 000 person years | N | CIN3+, rate per 100 000 person years | Cervical cancer, rate per 100 000 person years |
| Total | 517884 | 152.1 | 34.0 | 109009 | 314.5 | 20.1 |
| Year of birth  … 1932  1933-1946  1947-1956  1957-1966  1967-1976  1977-1982  1983-1988 | 43980  98551  90975  88290  87079  52300  56709 | 38.9  63.8  73.1  119.5  194.6  278.2  348.1 | 28.5  35.9  35.9  45.9  37.4  24.1  16.4 | 52300  56709 | 278.2  348.1 | 24.1  16.4 |
| Neoplasia (severest diagnosis)  CIN 1 (N87.0)  CIN2 (N87.1)  CIN3 (N87.2) or CIS(D06) None | 9332  11188  3622  493742 | 512.7  705.1  2242.2  118.3 | 46.9  63.1  159.6  32.3 | 4449  4907  1483  98170 | 720.1  895.8  2183.7  243.6 | 29.2  34.8  67.7  18.3 |
| Contraceptive coverage (systemic hormonal)  0  <0.10  0.10-0.40  0.40-0.60  0.60-1 | 393162  48307  41788  16330  18297 | 113.9  224.3  267.2  299.2  341.6 | 38.3  21.5  20.3  14.8  28.5 | 37069  22645  27024  11366  10905 | 266.8  306.7  328.8  351.1  420.2 | 25.0  17.4  13.4  24.6  16.3 |
| PAP test coverage  0  <0.20  0.20-0.40  0.40-0.80  0.80-1.00 | 216363  41820  92977  117520  49204 | 99.6  144.8  137.6  216.6  243.1 | 51.3  21.0  15.9  28.5  22.8 | 19092  10234  23890  39307  16486 | 182.3  277.8  285.1  373.2  394.9 | 19.9  17.4  11.4  23.9  25.6 |
| Proportion of period covered by health Insurance  0.01-0.50  0.50-0.80  0.80-0.99  1 | 14009  19632  53006  431237 | 211.5  249.5  233.7  135.0 | 75.7  63.2  42.4  30.2 | 4495  7229  23468  73817 | 286.4  360.4  333.4  305.8 | 31.2  37.1  27.1  15.5 |
| Number of births  0  1  2  3+ | 373442  51171  59851  33420 | 110.2  288.6  236.0  232.1 | 35.2  21.9  29.2  49.0 | 41565  34757  25748  6939 | 276.6  350.6  308.9  382.9 | 10.7  21.2  28.1  40.5 |
| Number of abortions  0  1  2  3+ | 467386  35858  9899  4741 | 137.0  271.6  323.0  308.7 | 34.8  25.5  25.7  42.9 | 80485  19557  5846  3121 | 293.3  363.5  429.4  343.2 | 18.6  22.1  26.3  32.7 |
| Smoking  Yes  No  No data | 13130  145525  359229 | 421.2  243.3  102.2 | 58.8  24.6  37.2 | 76736  24745  7528 | 516.0  311.1  264.9 | 49.7  18.0  17.5 |
| Education  Primary  Secondary  Tertiary  No data | 16427  92245  57488  351724 | 487.2  257.9  197.0  98.0 | 68.9  31.2  11.2  37.1 | 11290  41841  35152  20726 | 560.7  343.3  239.7  251.8 | 54.8  23.1  8.6  14.7 |
| HPV positive | 4638 | 484.9 | 38.8 | 2991 | 589.8 | 25.8 |
| HIV positive | 1096 | 938.8 | 175.2 | 878 | 1049.8 | 156.7 |
| Genital Chlamydia | 11280 | 459.8 | 29.5 | 7840 | 577.6 | 31.2 |
| Other STD | 56855 | 279.3 | 24.2 | 24893 | 416.3 | 19.5 |

**Supplement Table 3. Total and subgroup-specific number of women with CIN3+ and cervical cancer during the prediction period 2013-2017 (5-year timeframe)**

|  | **Cohort 1 (women born ≤1988)** | | | **Cohort 2 (women born 1977-1988)** | | |
| --- | --- | --- | --- | --- | --- | --- |
| Variable | N | CIN3+, rate per 100 000 person years | Cervical cancer, rate per 100 000 person years | N | CIN3+, rate per 100 000 person years | Cervical cancer, rate per 100 000 person years |
| Total | 517884 | 171.5 | 35.8 | 109009 | 355.8 | 22.1 |
| Year of birth  … 1932  1933-1946  1947-1956  1957-1966  1967-1976  1977-1982  1983-1988 | 43980  98551  90975  88290  87079  52300  56709 | 39.5  71.3  81.9  138.9  229.8  328.6  380.9 | 28.2  39.4  36.9  46.9  39.7  29.4  15.3 | 52300  56709 | 328.6  380.9 | 29.4  15.3 |
| Neoplasia (severest diagnosis)  CIN 1 (N87.0)  CIN2 (N87.1)  CIN3 (N87.2) or CIS(D06) None | 9332  11188  3622  493742 | 641.8  920.9  3220.7  125.0 | 54.8  81.4  175.4  33.5 | 4449  4907  1483  98170 | 923.5  1167.9  3124.7  253.7 | 36.9  46.5  91.5  19.3 |
| Contraceptive coverage (systemic hormonal)  0  <0.10  0.10-0.40  0.40-0.60  0.60-1 | 393162  48307  41788  16330  18297 | 127.4  255.8  312.1  322.9  407.6 | 39.7  22.6  22.7  18.6  34.3 | 37069  22645  27024  11366  10905 | 297.9  347.4  376.2  375.5  498.9 | 27.3  16.9  17.2  16.0  33.5 |
| PAP test coverage  0  <0.20  0.20-0.40  0.40-0.80  0.80-1.00 | 216363  41820  92977  117520  49204 | 101.5  174.8  158.7  251.4  294.6 | 49.8  24.9  19.2  32.7  25.1 | 19092  10234  23890  39307  16486 | 186.9  320.2  320.5  418.3  476.9 | 19.0  19.8  10.9  28.4  28.3 |
| Proportion of period covered by health Insurance  0.01-0.50  0.50-0.80  0.80-0.99  1 | 14009  19632  53006  431237 | 237.6  265.0  260.1  153.7 | 72.9  61.3  45.9  32.1 | 4495  7229  23468  73817 | 342.5  390.2  363.2  350.8 | 27.0  36.5  29.3  18.1 |
| Number of births  0  1  2  3+ | 373442  51171  59851  33420 | 121.9  327.4  281.2  271.0 | 36.2  23.7  34.8  52.0 | 41565  34757  25748  6939 | 305.3  392.9  363.2  445.1 | 11.2  22.7  33.0  43.9 |
| Number of abortions  0  1  2  3+ | 467386  35858  9899  4741 | 152.8  322.0  393.2  358.9 | 36.1  32.1  32.8  42.7 | 80485  19557  5846  3121 | 326.9  419.7  511.8  409.3 | 19.9  24.9  38.3  32.5 |
| Smoking  Yes  No  No data | 13130  145525  359229 | 494.0  281.9  112.9 | 68.4  27.1  38.2 | 76736  24745  7528 | 605.9  354.5  284.3 | 54.3  19.8  19.6 |
| Education  Primary  Secondary  Tertiary  No data | 16427  92245  57488  351724 | 563.9  298.5  229.8  108.1 | 67.1  35.9  13.3  38.1 | 11290  41841  35152  20726 | 640.8  393.1  273.3  267.2 | 48.9  28.1  10.3  15.6 |
| HPV positive | 4638 | 554.0 | 35.2 | 2991 | 662.6 | 27.3 |
| HIV positive | 1096 | 1065.4 | 193.7 | 878 | 1188.3 | 194.0 |
| Genital Chlamydia | 11280 | 513.9 | 32.5 | 7840 | 642.9 | 33.8 |
| Other STD | 56855 | 326.7 | 27.1 | 24893 | 476.7 | 17.9 |

**Supplement Table 4. Cox regression model performance among Cohort 1 and Cohort 2 populations fitted three sets of predictors**

| Sample | All |  |  |  |  |  | Young |  |  |  |  |  |
| --- | --- | --- | --- | --- | --- | --- | --- | --- | --- | --- | --- | --- |
| Outcome | Cancer |  |  | CIN3+ |  |  | Cancer |  |  | CIN3+ |  |  |
| Selection of explanatory variables | All ^1^ | EHIF^2^ | LASSO^3^ | All | EHIF | LASSO | All | EHIF | LASSO | All | EHIF | LASSO |
| Harrell C | 0.681 (0.667 - 0.695) | 0.662 (0.647 - 0.677) | 0.681 (0.667 - 0.695) | 0.738 (0.731 - 0.745) | 0.733 (0.726 - 0.74) | 0.738 (0.731 - 0.745) | 0.721 (0.684 - 0.759) | 0.639 (0.599 - 0.679) | 0.721 (0.684 - 0.759) | 0.686 (0.675 - 0.697) | 0.666 (0.655 - 0.677) | 0.685 (0.675 - 0.696) |
| Uno C | 0.682 (0.668 - 0.697) | 0.663 (0.648 - 0.678) | 0.682 (0.668 - 0.697) | 0.736 (0.729 - 0.743) | 0.73 (0.724 - 0.737) | 0.736 (0.729 - 0.743) | 0.721 (0.684 - 0.759) | 0.639 (0.599 - 0.679) | 0.721 (0.684 - 0.759) | 0.686 (0.675 - 0.696) | 0.666 (0.654 - 0.677) | 0.685 (0.675 - 0.696) |
| Harrell C cross-validated | 0.673 (0.658 - 0.687) | 0.655 (0.64 - 0.67) | 0.673 (0.658 - 0.687) | 0.737 (0.73 - 0.743) | 0.731 (0.725 - 0.738) | 0.737 (0.73 - 0.743) | 0.671 (0.631 - 0.71) | 0.593 (0.552 - 0.635) | 0.671 (0.631 - 0.71) | 0.682 (0.671 - 0.693) | 0.664 (0.653 - 0.675) | 0.682 (0.672 - 0.693) |
| Uno C cross-validated | 0.674 (0.659 - 0.688) | 0.656 (0.641 - 0.671) | 0.674 (0.659 - 0.688) | 0.734 (0.727 - 0.741) | 0.729 (0.722 - 0.736) | 0.734 (0.727 - 0.741) | 0.671 (0.631 - 0.71) | 0.593 (0.552 - 0.635) | 0.671 (0.631 - 0.71) | 0.682 (0.671 - 0.693) | 0.664 (0.653 - 0.675) | 0.682 (0.671 - 0.693) |
| AUROC 5 years | 0.662 | 0.645 | 0.662 | 0.739 | 0.734 | 0.739 | 0.666 | 0.629 | 0.666 | 0.705 | 0.685 | 0.705 |
| AUROC 8 years | 0.675 | 0.657 | 0.675 | 0.721 | 0.715 | 0.721 | 0.671 | 0.594 | 0.671 | 0.683 | 0.665 | 0.684 |
| Observed/expected 5 years | 1 (0.937 - 1.068) | 1 (0.937 - 1.068) | 1 (0.937 - 1.068) | 1.013 (0.983 - 1.044) | 1.013 (0.983 - 1.043) | 1.013 (0.983 - 1.044) | 0.997 (0.833 - 1.194) | 0.999 (0.834 - 1.195) | 0.997 (0.833 - 1.194) | 0.996 (0.952 - 1.042) | 0.996 (0.952 - 1.042) | 0.996 (0.953 - 1.042) |
| Observed/expected 8 years | 1 (0.948 - 1.055) | 1 (0.948 - 1.056) | 1 (0.948 - 1.055) | 1.027 (1.001 - 1.053) | 1.026 (1.001 - 1.053) | 1.027 (1.001 - 1.053) | 0.996 (0.858 - 1.157) | 0.998 (0.859 - 1.159) | 0.996 (0.858 - 1.157) | 0.997 (0.96 - 1.036) | 0.997 (0.96 - 1.035) | 0.997 (0.96 - 1.036) |
| Calibration slope | 0.922 (0.844 - 1.001) | 0.955 (0.867 - 1.044) | 0.922 (0.844 - 1.001) | 0.993 (0.971 - 1.015) | 0.995 (0.972 - 1.018) | 0.993 (0.971 - 1.015) | 0.761 (0.588 - 0.934) | 0.795 (0.546 - 1.045) | 0.761 (0.588 - 0.934) | 0.985 (0.939 - 1.031) | 0.991 (0.943 - 1.039) | 0.987 (0.941 - 1.032) |

^1^ all available predictors were used

^2^ only predictors from EHIF used (exl smoking, education, number of births)

^3^LASSO method was used to select predictors


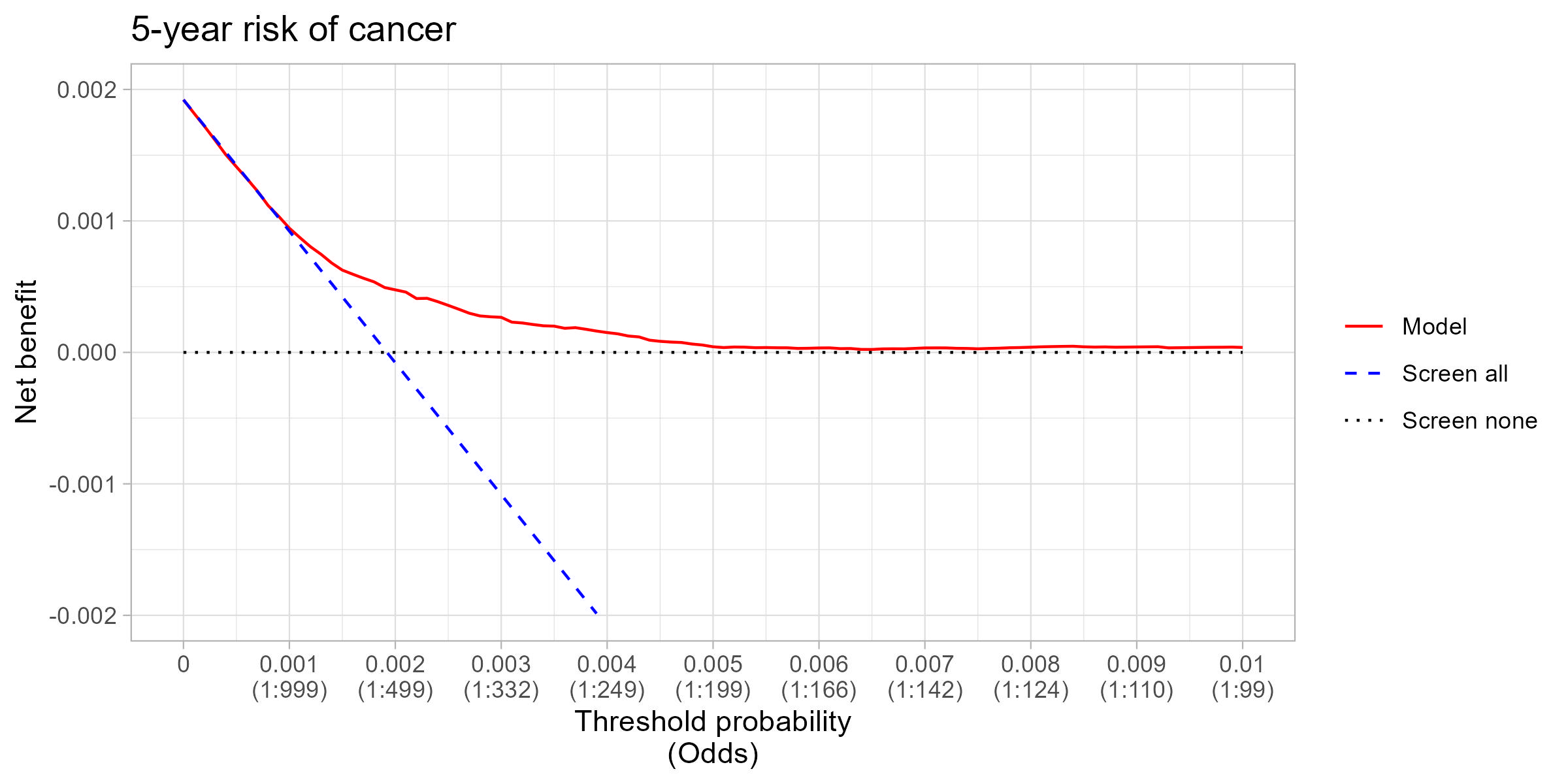


Sup Figure 1 Decision curve analysis at 5 years for the cervical cancer risk prediction model among women aged 30-65 y using LASSO Cox model chosen predictors to guide decisions at cervical cancer screening program


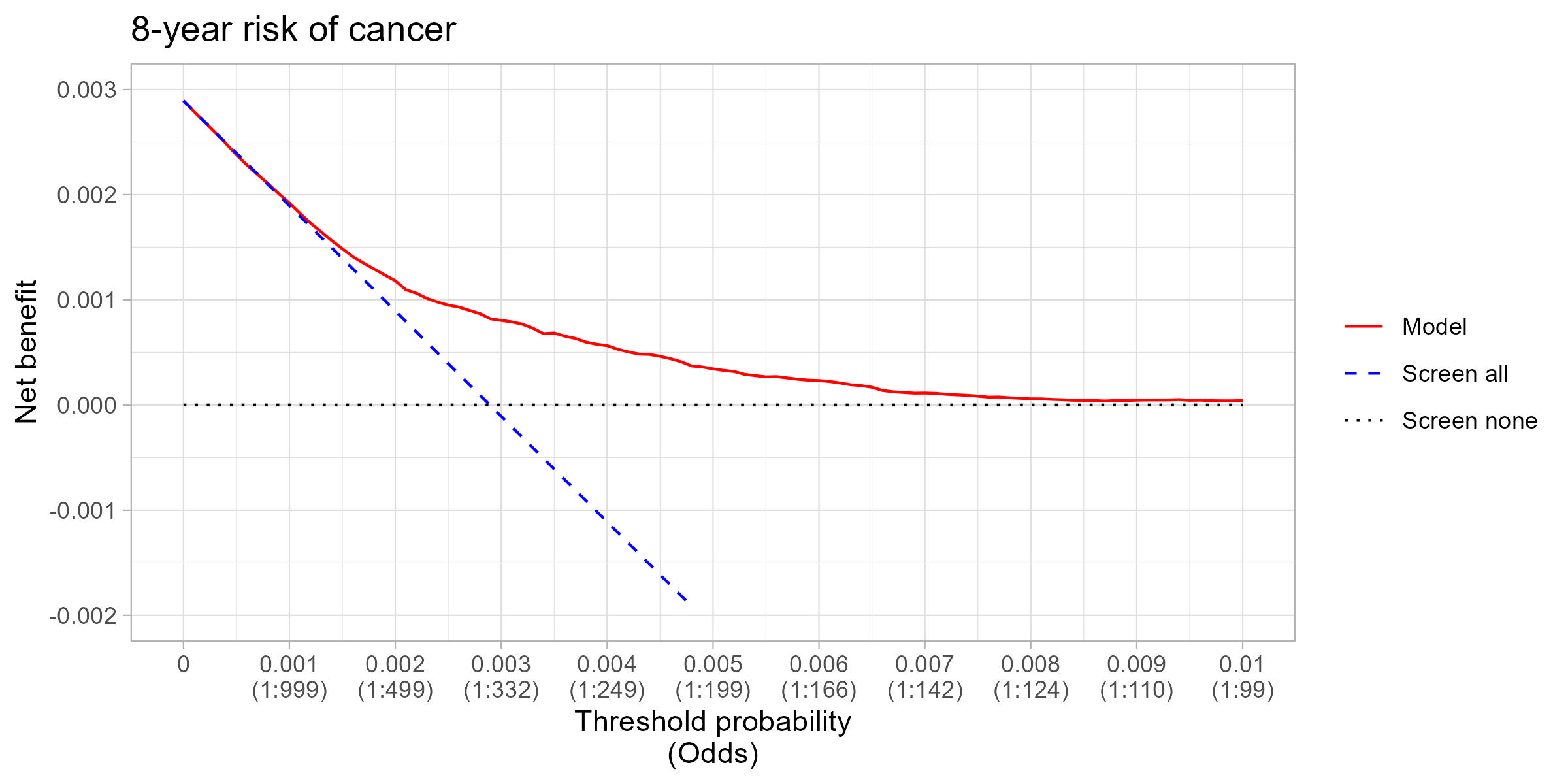


Sup Figure 2 Decision curve analysis at 8 years for the cervical cancer risk prediction model among women aged 30-65 y using LASSO Cox model chosen predictors to guide decisions at cervical cancer screening program.


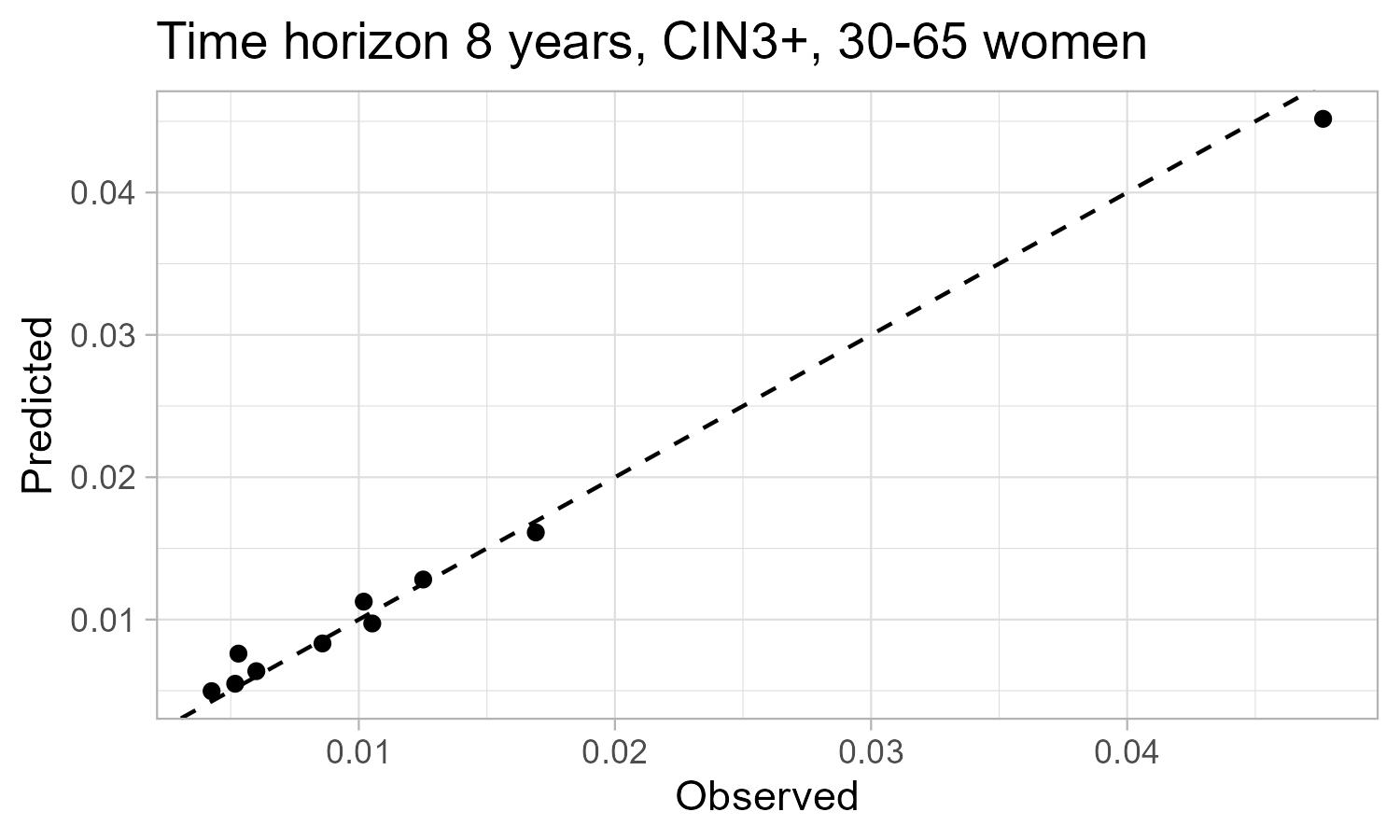


Sup Fig 3. Calibration plot at 8 years risk prediction model for the CIN3+ among women aged 30-65 y


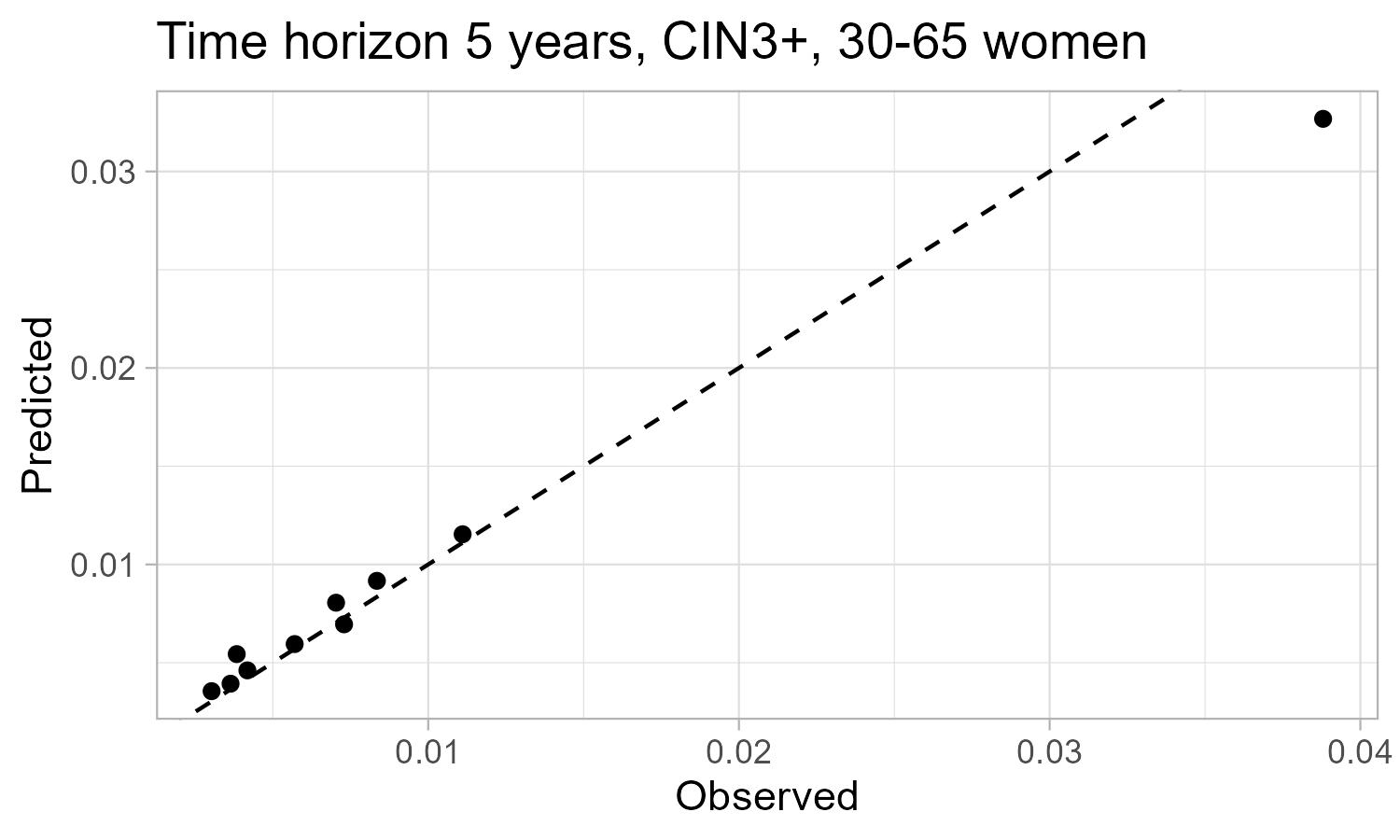


Sup Fig 4. Calibration plot at 5 years risk prediction model for the CIN3+ among women aged 30-65 y


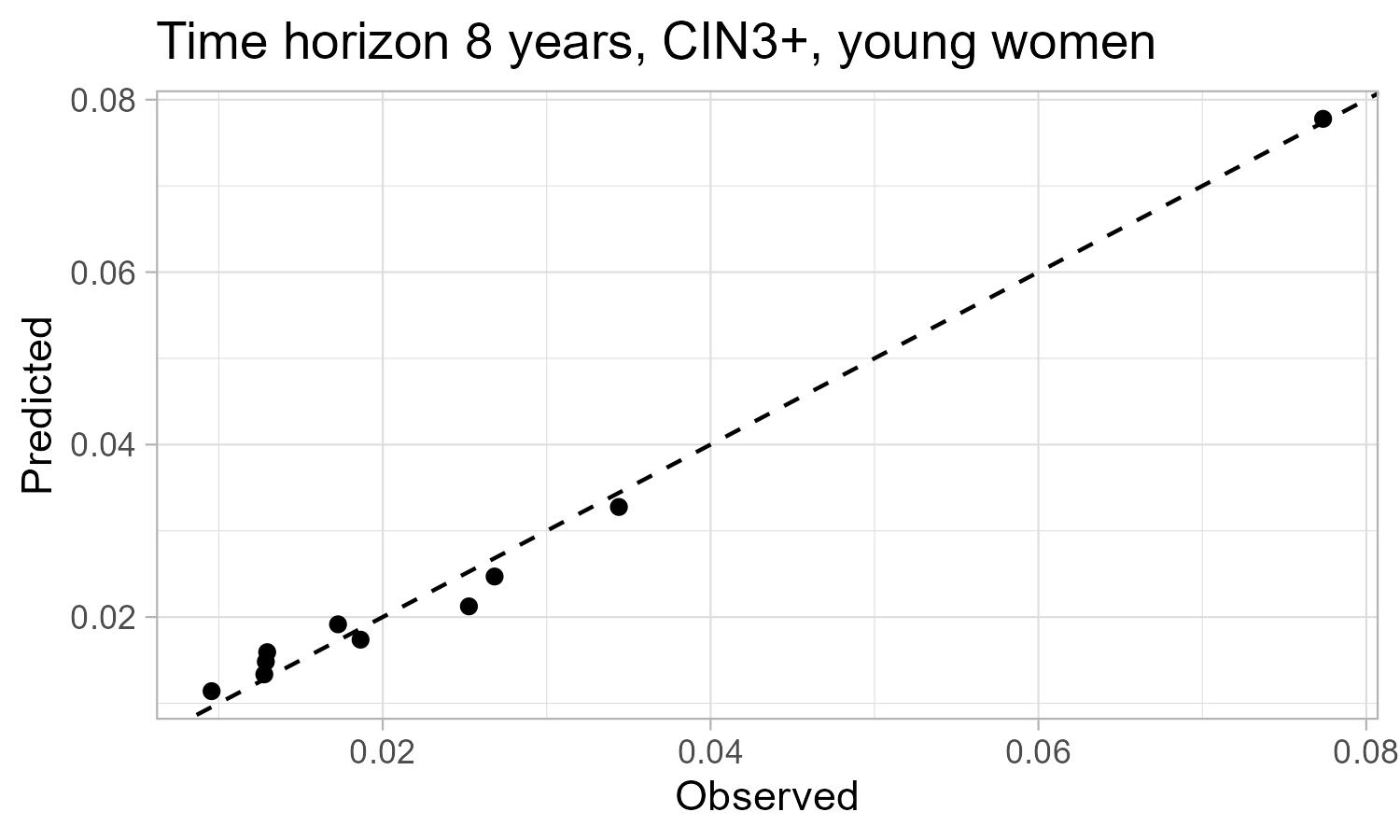


Sup Fig 5. Calibration plot at 8 years risk prediction model for the CIN3+ among Cohort 2 women (women born 1977-1988)


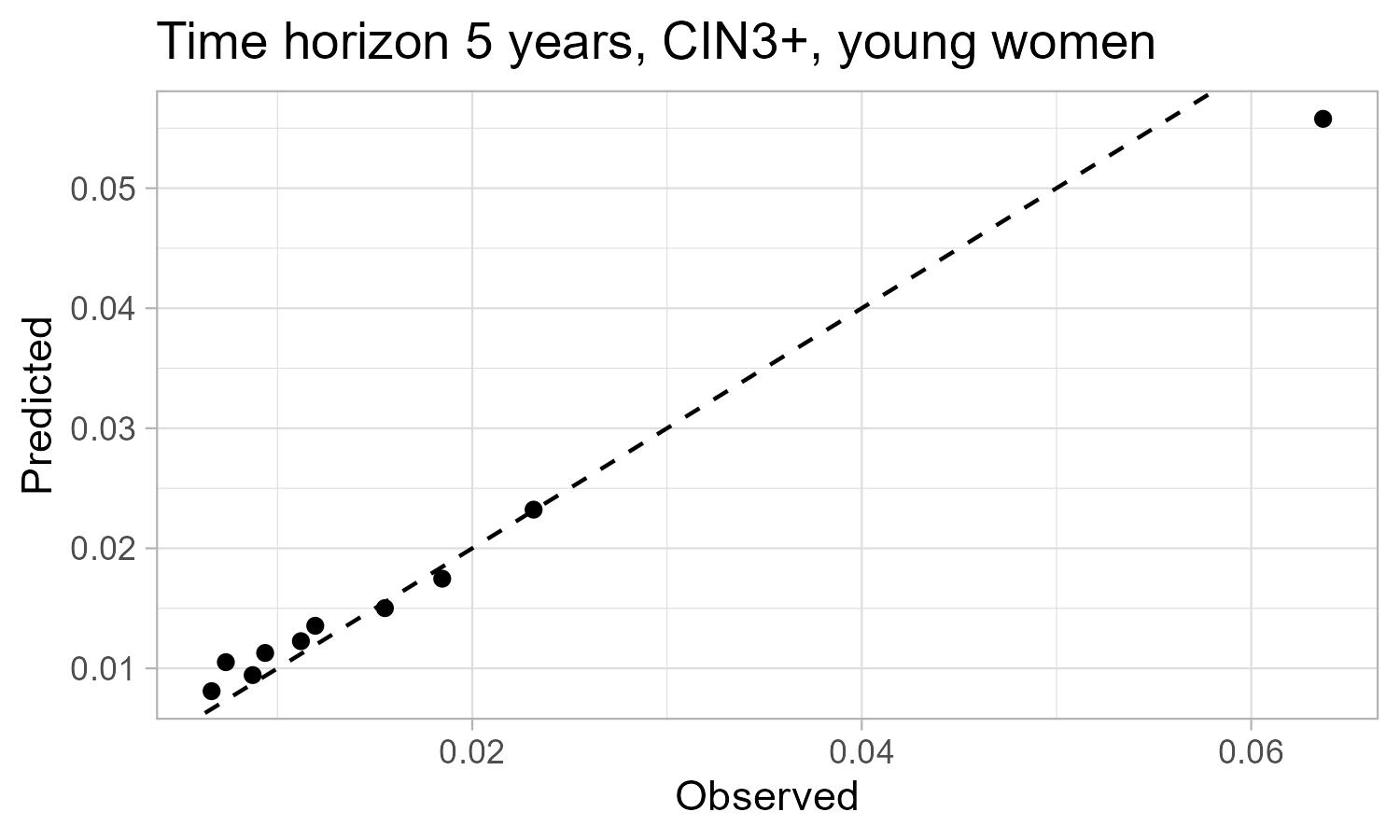


Sup Fig 6. Calibration plot at 5 years risk prediction model for the CIN3+ among Cohort 2 women (women born 1977-1988)


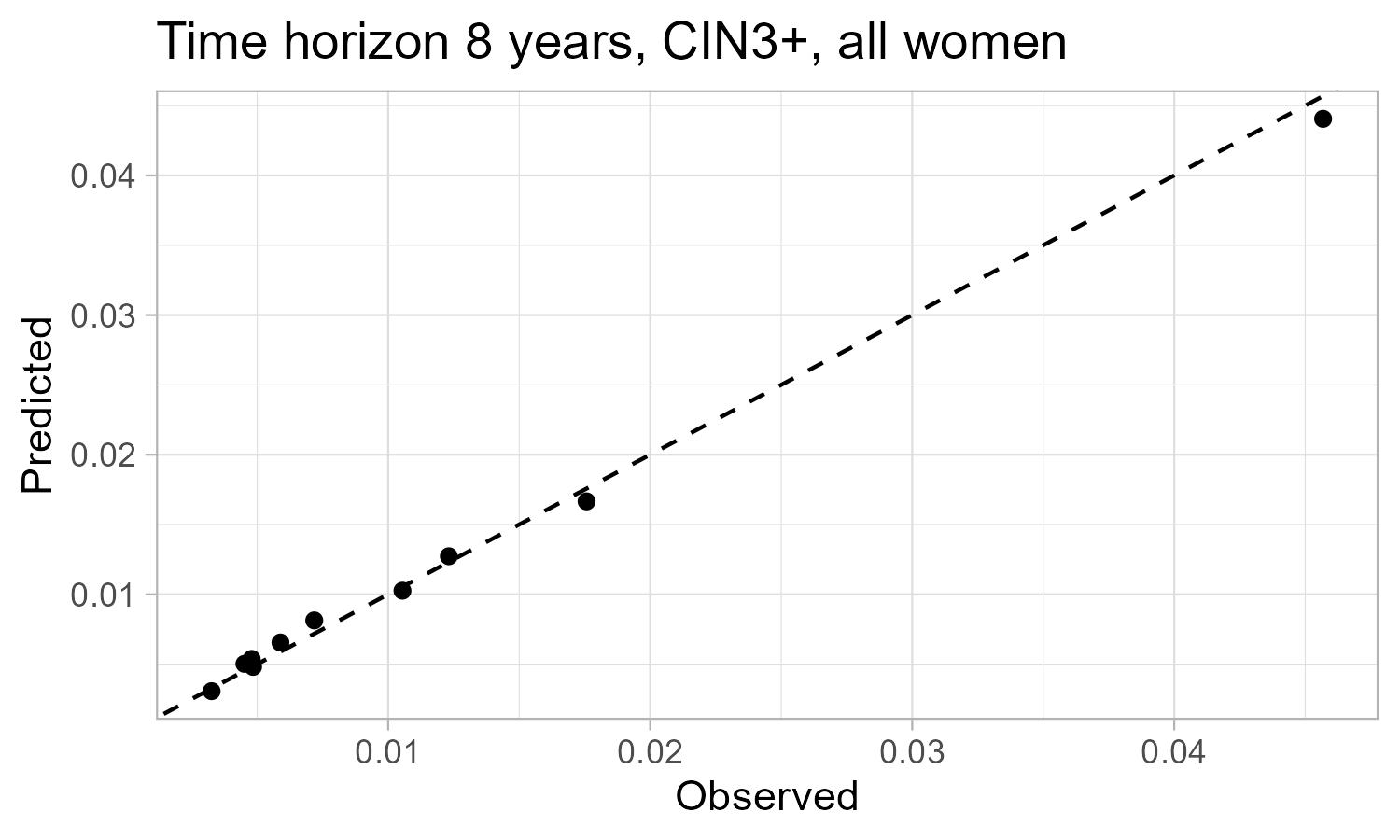


Sup Fig 7. Calibration plot at 8 years risk prediction model for the CIN3+ among Cohort 1 women (women born ≤1988)


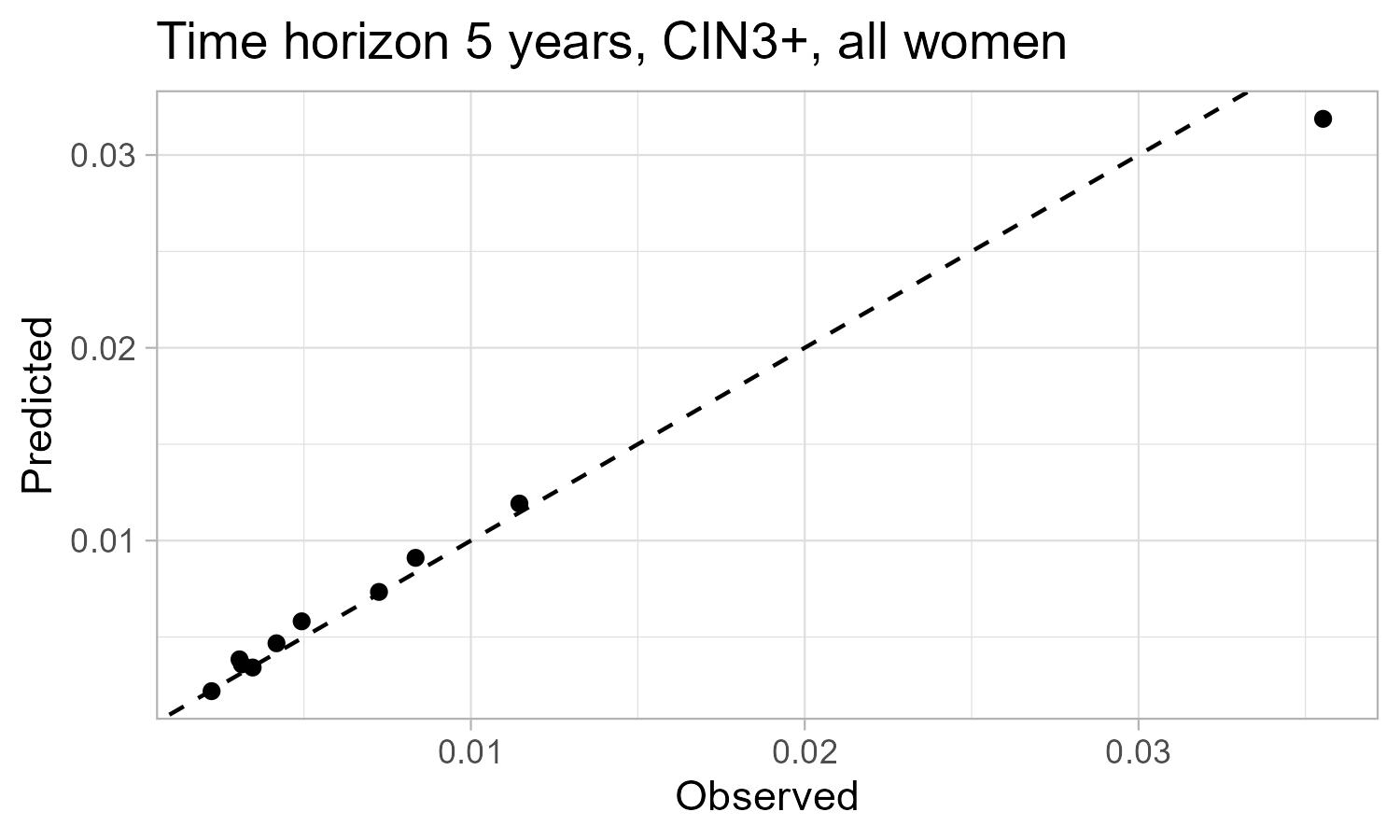


Sup Fig 8. Calibration plot at 5 years risk prediction model for the CIN3+ among Cohort 1 women (women born ≤1988)


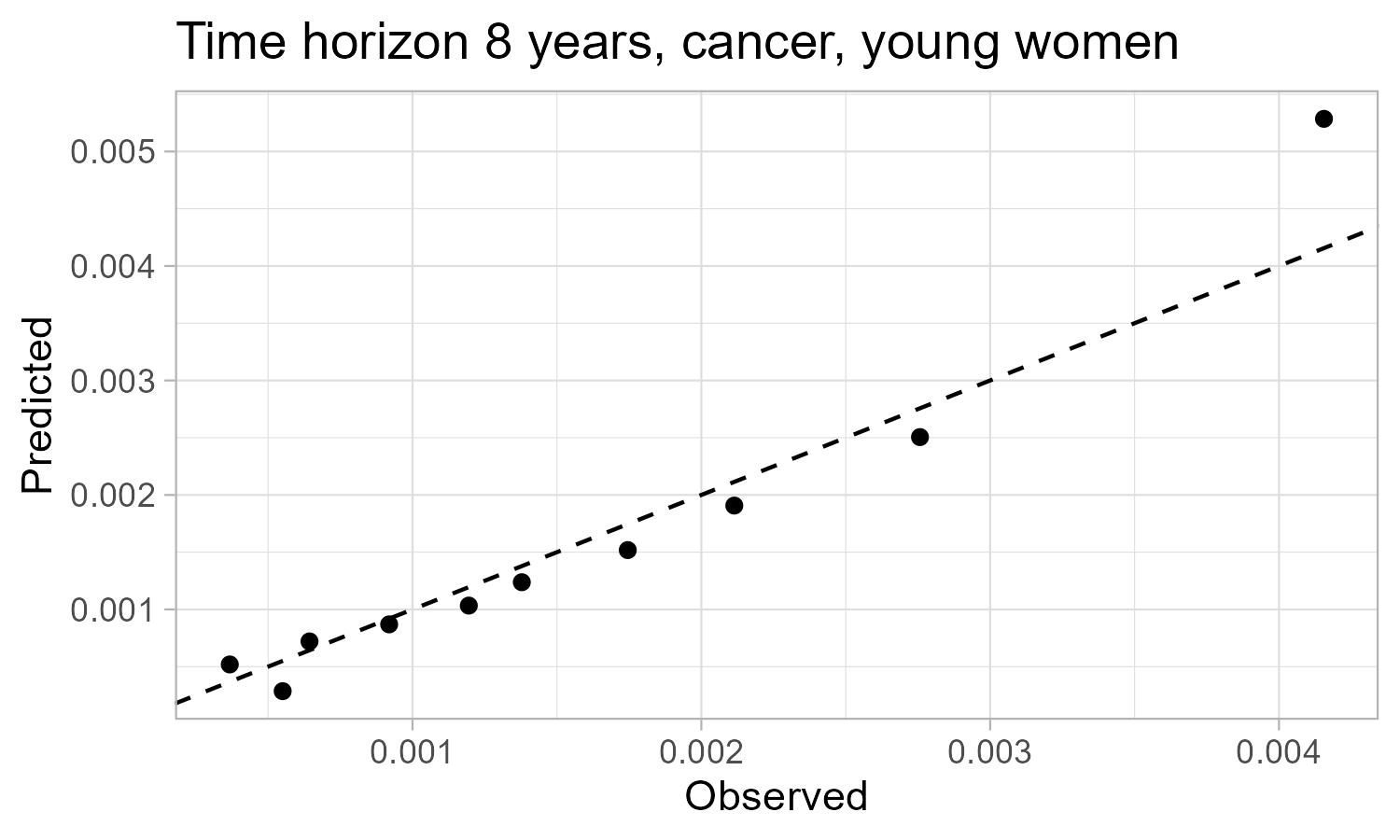


Sup Fig 9. Calibration plot at 8 years risk prediction model for cervical cancer among Cohort 2 women (women born 1977-1988)


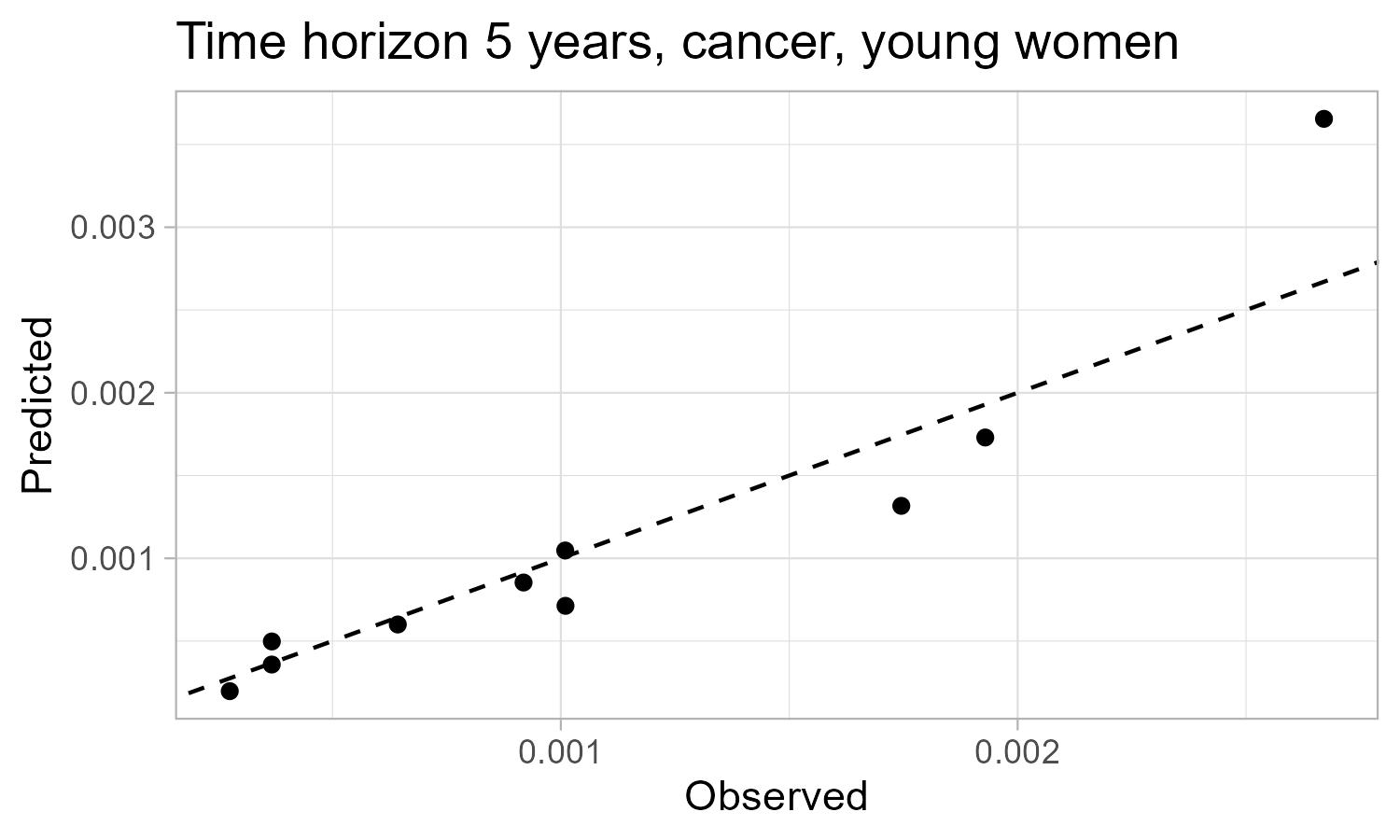


Sup Fig 10. Calibration plot at 5 years risk prediction model for cervical cancer among Cohort 2 women (women born 1977-1988)


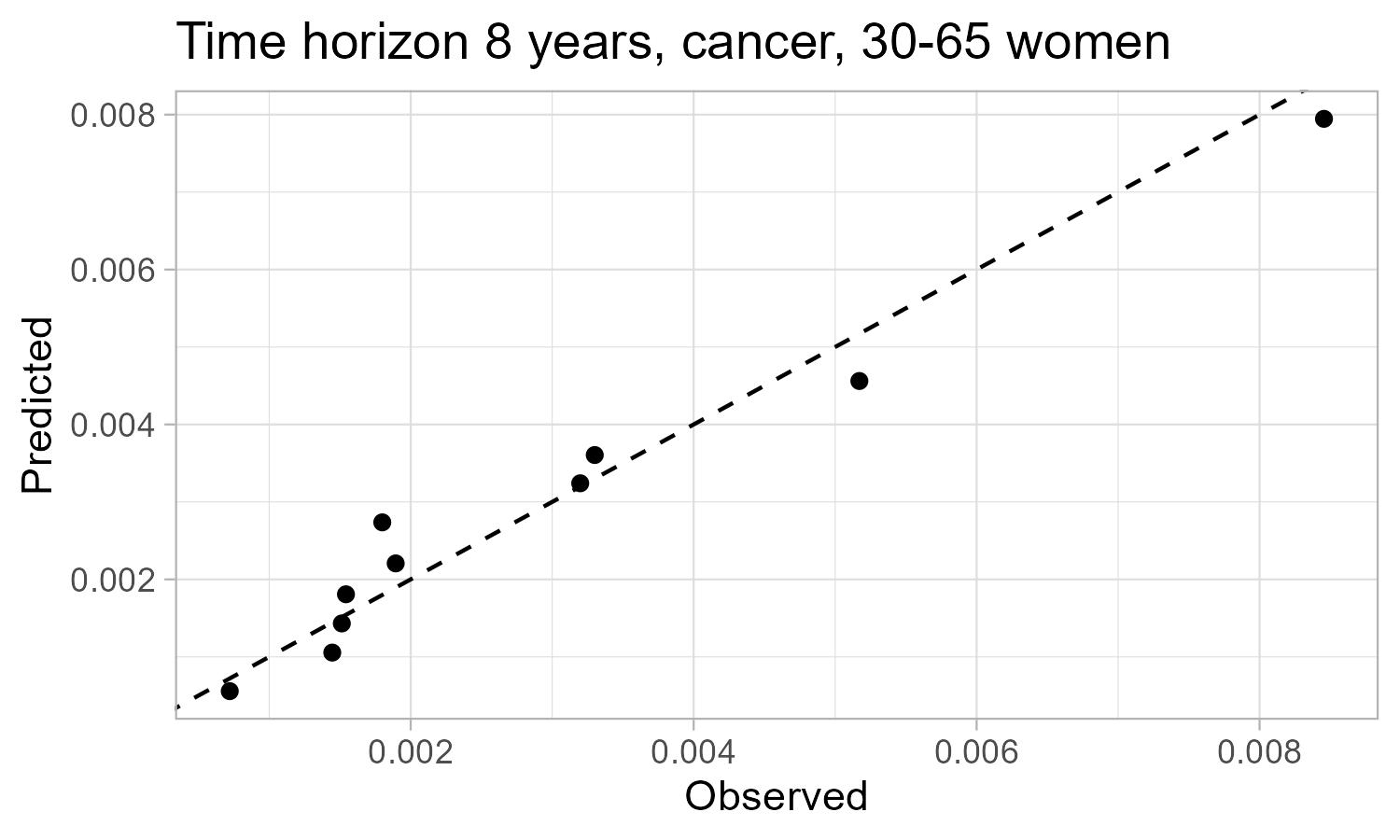


Sup Fig 11. Calibration plot at 8 years risk prediction model for cervical cancer among women aged 30-65 y


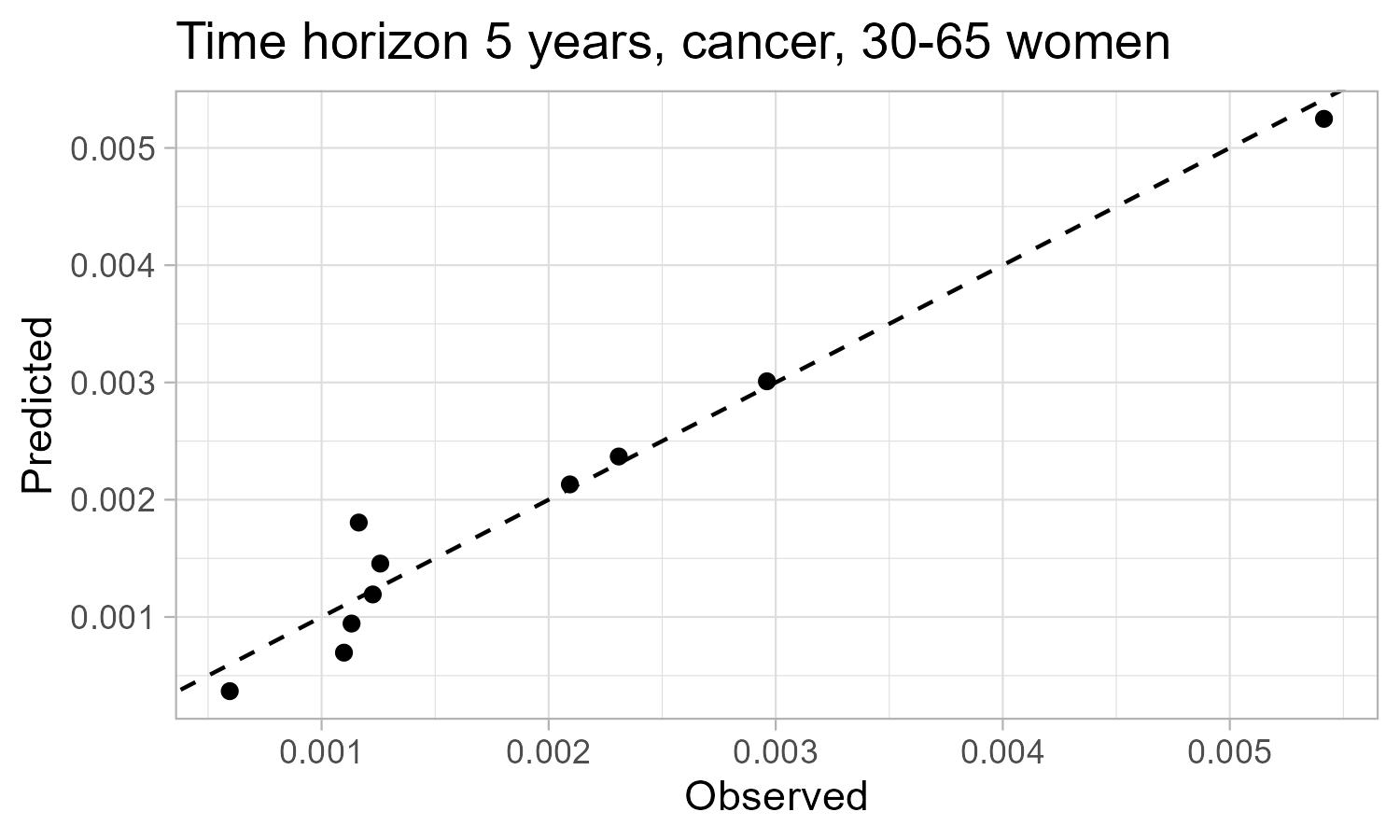


Sup Fig 12. Calibration plot at 5 years risk prediction model for cervical cancer among women aged 30-65 y


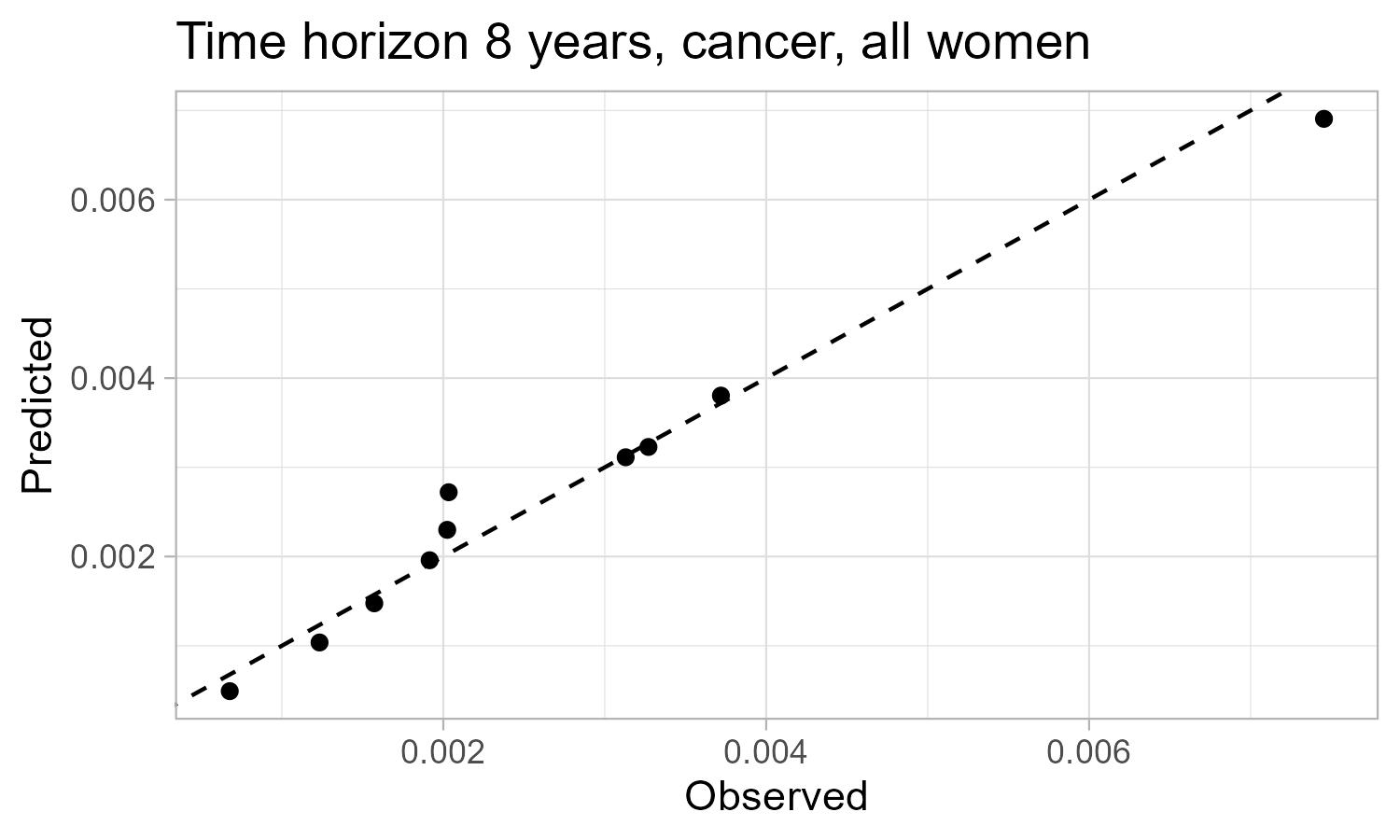


Sup Fig 13. Calibration plot at 8 years risk prediction model for cervical cancer among Cohort 1 women (women born ≤1988)


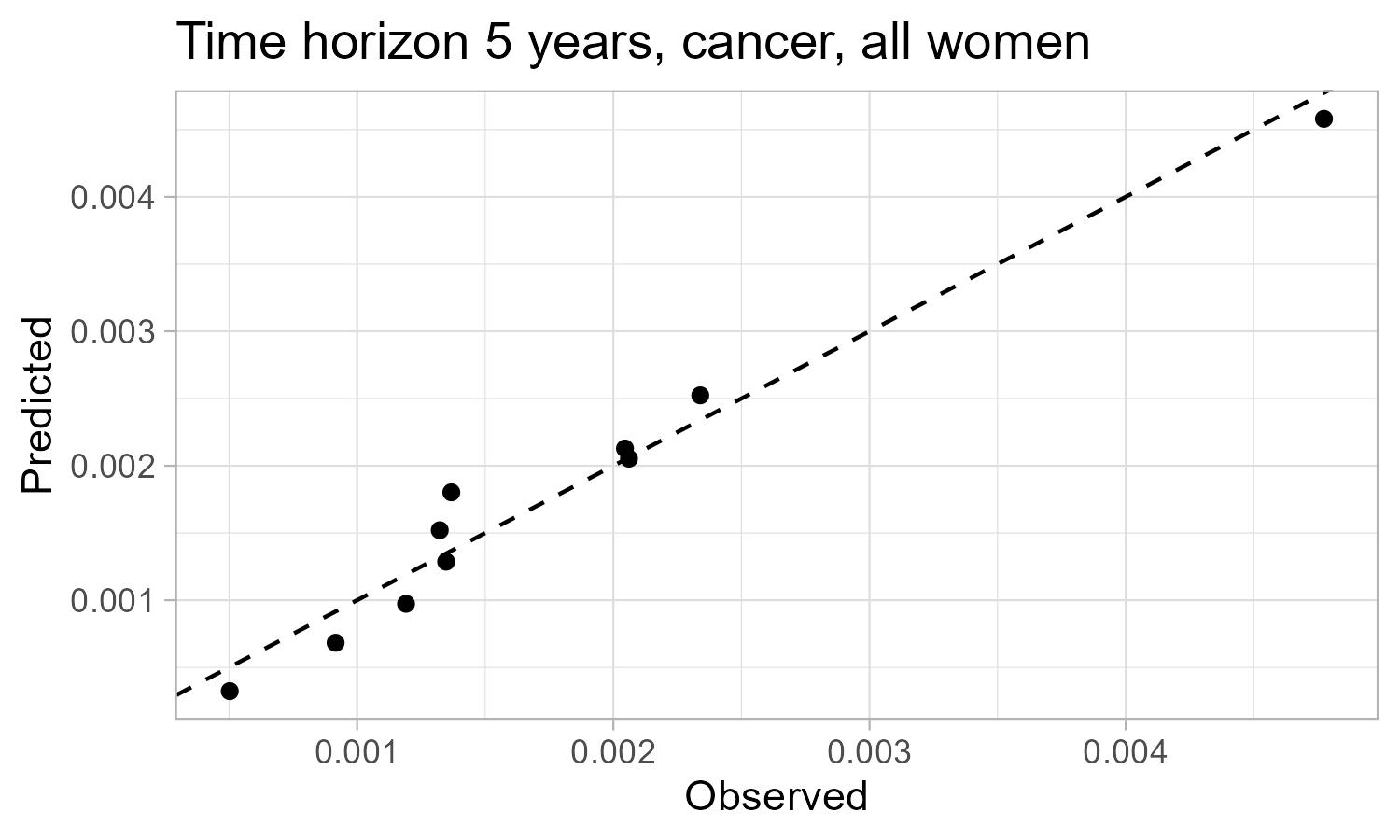


Sup Fig 14. Calibration plot at 5 years risk prediction model for cervical cancer among Cohort 1 women (women born ≤1988)

Supplement Table 4. Final Cox proportional hazards model (selected by LASSO) coefficients

|  | All |  | Young |  |
| --- | --- | --- | --- | --- |
|  | Cancer | CIN3+ | Cancer | CIN3+ |
| Previous CIN1 | 0.995 | 1.134 | 0.500 | 0.977 |
| Previous CIN2 | 1.200 | 1.442 | 0.559 | 1.162 |
| Previous CIN3 | 1.980 | 2.590 | 1.018 | 2.047 |
| birthyear upto1932 | 0.000 | 0.000 |  |  |
| birthyear 1933-1946 | 0.301 | 0.550 |  |  |
| birthyear 1947-1956 | 0.350 | 0.657 |  |  |
| birthyear 1957-1966 | 0.721 | 1.067 |  |  |
| birthyear 1967-1976 | 0.627 | 1.423 |  |  |
| birthyear 1977-1982 | 0.483 | 1.730 | 0.000 | 0.000 |
| birthyear 1983-1988 | 0.271 | 1.975 | -0.271 | 0.220 |
| Abortions | 0.012 | -0.024 | -0.052 | -0.020 |
| Births: 0 | 0.000 | 0.000 | 0.000 | 0.000 |
| Births: 1 | 1.043 | 0.018 | 0.713 | 0.000 |
| Births: 2 | 1.424 | 0.052 | 1.027 | -0.057 |
| Births: 3+ | 1.740 | 0.170 | 1.125 | 0.047 |
| Births: NA | 1.033 | 0.269 |  |  |
| smoking: No | 0.000 | 0.000 | 0.000 | 0.000 |
| smoking: UKN | 0.444 | 0.131 | 0.482 |  |
| smoking: Yes | 0.297 | 0.239 | 0.369 | 0.201 |
| education: Primary | 0.000 | 0.000 | 0.000 | 0.000 |
| education: Secondary | -0.601 | -0.349 | -0.638 | -0.361 |
| education: Tertiary | -1.337 | -0.682 | -1.423 | -0.715 |
| education: UKN | -0.534 | -0.695 | -0.723 | -0.578 |
| chlamydia | 0.204 | 0.326 | 0.420 | 0.388 |
| HIV | 1.382 | 0.719 | 1.486 | 0.801 |
| HPV | 0.369 | 0.186 | 0.189 | 0.223 |
| Other STD | -0.223 | 0.028 | -0.225 | 0.033 |
| PAP coverage | -1.378 | -0.236 | -0.066 | 0.155 |
| Contraceptive coverage | 0.177 | 0.390 | 0.531 | 0.497 |
| Insured | -1.007 | -0.487 | -0.974 | -0.312 |

Supplement Table 5. LR test-statistics and p-values for individual variables for the final models

|  | All |  |  |  | Young |  |  |  |
| --- | --- | --- | --- | --- | --- | --- | --- | --- |
|  | Cancer |  | CIN3+ |  | Cancer |  | CIN3+ |  |
|  | LR | p | LR | p | LR | p | LR | p |
| Abortions | 0.0 | 0.841 | 1.5 | 0.227 | 0.4 | 0.551 | 0.8 | 0.380 |
| Chlamydia | 0.9 | 0.333 | 33.7 | 0.000 | 2.6 | 0.110 | 39.7 | 0.000 |
| HIV | 16.9 | 0.000 | 29.7 | 0.000 | 13.1 | 0.000 | 31.8 | 0.000 |
| HPV | 1.6 | 0.203 | 5.3 | 0.021 | 0.2 | 0.665 | 5.8 | 0.016 |
| Other STD | 10.9 | 0.001 | 2.2 | 0.141 | 3.9 | 0.049 | 2.0 | 0.155 |
| PAP coverage | 141.6 | 0.000 | 22.2 | 0.000 | 0.1 | 0.822 | 4.2 | 0.039 |
| Contraceptive coverage | 0.8 | 0.384 | 38.3 | 0.000 | 2.6 | 0.107 | 40.2 | 0.000 |
| Insurance | 36.9 | 0.000 | 27.8 | 0.000 | 4.4 | 0.037 | 5.1 | 0.024 |
| Previous CIN1 | 23.5 | 0.000 | 310.5 | 0.000 | 2.0 | 0.161 | 157.1 | 0.000 |
| Previous CIN2 | 50.2 | 0.000 | 729.8 | 0.000 | 3.1 | 0.079 | 280.4 | 0.000 |
| Previous CIN3 | 85.9 | 0.000 | 1745.5 | 0.000 | 5.1 | 0.024 | 499.7 | 0.000 |
| 1933-1946 | 5.1 | 0.023 | 26.3 | 0.000 |  |  |  |  |
| 1947-1956 | 6.8 | 0.009 | 38.9 | 0.000 |  |  |  |  |
| 1957-1966 | 30.8 | 0.000 | 119.7 | 0.000 |  |  |  |  |
| 1967-1976 | 17.3 | 0.000 | 204.9 | 0.000 |  |  |  |  |
| 1977-1982 | 5.8 | 0.016 | 255.9 | 0.000 |  |  |  |  |
| 1983-1988 | 1.4 | 0.235 | 328.6 | 0.000 | 2.7 | 0.101 | 28.4 | 0.000 |
| smoking: UKN | 3.8 | 0.050 | 2.6 | 0.107 | 1.9 | 0.173 |  |  |
| smoking: Yes | 3.7 | 0.056 | 17.8 | 0.000 | 2.4 | 0.118 | 8.7 | 0.003 |
| education: Secondary | 18.4 | 0.000 | 46.9 | 0.000 | 9.8 | 0.002 | 37.9 | 0.000 |
| education: Tertiary | 51.9 | 0.000 | 129.5 | 0.000 | 27.4 | 0.000 | 111.5 | 0.000 |
| education: UKN | 1.5 | 0.216 | 37.4 | 0.000 | 1.9 | 0.171 | 58.0 | 0.000 |
| Births: 1 | 12.2 | 0.000 | 0.1 | 0.763 | 4.8 | 0.028 |  |  |
| Births: 2 | 23.6 | 0.000 | 0.7 | 0.407 | 9.6 | 0.002 | 1.3 | 0.261 |
| Births: 3+ | 33.2 | 0.000 | 5.2 | 0.022 | 7.9 | 0.005 | 0.3 | 0.555 |
| Births: NA | 19.6 | 0.000 | 12.5 | 0.000 |  |  |  |  |

The likelihood ratio statistic is calculated by comparing the log-likelihood of the final model and the log-likelihood of the model excluding the variable under consideration
